# Hollowing Out Nanofibers for Enhanced Visual Brightness and More Effective Solar Heating

**DOI:** 10.1002/smll.76477

**Published:** 2026-10-27

**Authors:** Minghong Wu, Yuxuan Meng, Huajian Ji, Jianlong He, Min Hao, Younan Xia

**Affiliations:** ^1^ School of Chemical and Biomolecular Engineering Georgia Institute of Technology Atlanta Georgia USA; ^2^ School of Chemistry and Biochemistry Georgia Institute of Technology Atlanta Georgia USA; ^3^ The Wallace H. Coulter Department of Biomedical Engineering Georgia Institute of Technology and Emory University Atlanta Georgia USA; ^4^ Department of Materials Science and Engineering Johns Hopkins University Baltimore Maryland USA; ^5^ Department of Biomedical Engineering Johns Hopkins University Baltimore Maryland USA

**Keywords:** heat dissipation, hollow nanofibers, light backscattering, solar heating, thermal management

## Abstract

The temperature rise of a solar‐heating textile is controlled by both generation and dissipation of heat. However, current designs mainly focus on increasing light absorption to generate more heat, often darkening the material while leaving thermal management largely overlooked. Herein, hollowing the nanofibers in a nonwoven mat produces a counterintuitive “brighter‐yet‐hotter” advantage over their solid (core–shell) counterparts. The polyacrylonitrile/polypyrrole double‐shelled tubular nanofibers are fabricated by selectively removing the poly(ε‐caprolactone) core from core–shell nanofibers. At outer and inner diameters of about 320 and 160 nm, the hollow nanofibers enhance backscattering in the visible range, making the mat brighter. Under 1 sun irradiation, the mat of hollow nanofibers shows a temperature rise of 28°C, which is 12% greater than that of the paired solid counterpart. Thermal modeling indicates that the higher temperature rise originates from the internal channels, which disrupt heat propagation across the fibers and lower the rate of heat dissipation. Side‐by‐side comparisons under simulated and outdoor sunlight show that the higher‐temperature region consistently appears on the mat of hollow nanofibers. This work highlights the promise of hollow nanofibers as a structural platform for optical and thermal management in solar‐heating textiles.

## Graphical abstract

A solar‐heating mat of hollow nanofibers can be brighter yet hotter than its paired solid counterpart at suitable fiber dimensions. The hollow nanofibers enhance visible backscattering and suppress heat dissipation. Indoor and outdoor side‐by‐side tests show higher solar‐heating temperatures for the hollow mat, demonstrating hollowing as a structural strategy for optical and thermal management in solar‐heating textiles.

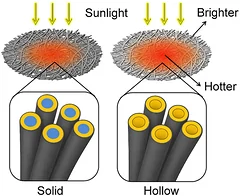

## 1 Introduction

Solar‐heating materials undergo temperature rise upon illumination by converting the absorbed light into heat [1, 2, 3]. Increasing light absorption and light‐to‐heat conversion efficiency are two major strategies for improving the solar‐heating performance, typically accomplished by selecting efficient photothermal fillers [4, 5], increasing the loading of the fillers [6, 7], and/or optimizing the absorption spectra [8, 9]. These absorption‐centered strategies, which often add fabrication complexity and cost [10, 11], tend to darken the material [12, 13] and hinder commercial adoption. Therefore, brightness and temperature rise are commonly considered a trade‐off in solar heating [14]. It is worth noting that temperature rise is not determined by heat generation alone. The generated heat is continuously dissipated through heat conduction within the material, heat convection with the surrounding environment, and surface thermal radiation [15, 16, 17]. Compared with the extensive efforts devoted to light absorption, structural regulation of these dissipation pathways remains largely unexplored in solar‐heating materials.

Hollow structures offer a promising platform for thermal management as the internal cavities and solid–air interfaces can alter heat‐transfer pathways [18, 19]. The same mechanism is also widely exploited by animals, where hollow hairs are used to regulate heat exchange between their bodies and the surrounding environment [20, 21]. When internal cavities are introduced into solar‐heating materials, however, their actual roles become more complex because the cavities can simultaneously redistribute the incident light through additional optical interfaces and alter the dissipation pathways of the heat [22, 23]. This coupling is particularly strong in fibrous solar‐heating materials, where multiple scattering in the porous fiber network lengthens the optical path and promotes light absorption [24], darkening solar‐heating textiles. The same fiber network also carries the generated heat, so any change to the fiber structure acts on the optical and thermal responses at the same time. Therefore, there is a pressing need to understand how the internal channels in individual fibers and the pores among the fibers work together to determine the visual brightness and solar‐heating performance.

In this work, we demonstrate that hollow nanofibers in a nonwoven mat can be engineered to increase both visual brightness and solar‐heating temperature rise when benchmarked against their solid counterparts. Core–shell nanofibers are first fabricated by coaxially electrospinning poly(ε‐caprolactone) (PCL) and polyacrylonitrile (PAN), followed by coating with polypyrrole (PPy) to yield a parent mat of PCL@PAN/PPy solid nanofibers. For meaningful comparison, paired mats of solid and hollow nanofibers are cut from the same parent mat to minimize sample‐to‐sample variation. Half of the mat is kept as a solid control while the other half is converted into PAN/PPy hollow nanofibers by selectively dissolving the PCL core. With specific inner and outer diameters, the hollow nanofibers can increase visible backscattering and enhance diffuse reflectance. The internal channels also disrupt heat propagation through the fibrous network, allowing the hollow sample to achieve a higher temperature rise upon solar illumination. Collectively, this work aims to establish the principle to engineer hollow nanofibers for regulating both optical appearance and thermal response in fibrous solar‐heating materials.

## 2 Results and Discussion

### 2.1 Fabrication of Paired Mats of Solid and Hollow Nanofibers

The fabrication of paired mats of solid and hollow nanofibers is illustrated in Figure 1A. We first prepared PCL@PAN core–shell nanofibers by coaxial electrospinning, in which the coaxial spinneret delivered PCL and PAN solutions as the core and shell, respectively, to produce a mat of uniform nanofibers. After immersion in an aqueous solution of pyrrole and FeCl_3_ for 6 h of polymerization, the mat changed appearance from white to gray, indicating PPy coating on the fiber's surface for the generation of PCL@PAN/PPy solid fibers (Figure S1). The paired samples were obtained by cutting the mat into multiple pieces, with half of them kept as solid controls. The other half was soaked in chloroform to selectively remove the PCL core, yielding PAN/PPy hollow nanofibers (Figure S2). Because both halves came from the same mat, their optical and thermal responses could be directly compared with minimal sample‐to‐sample variation.

**FIGURE 1:**
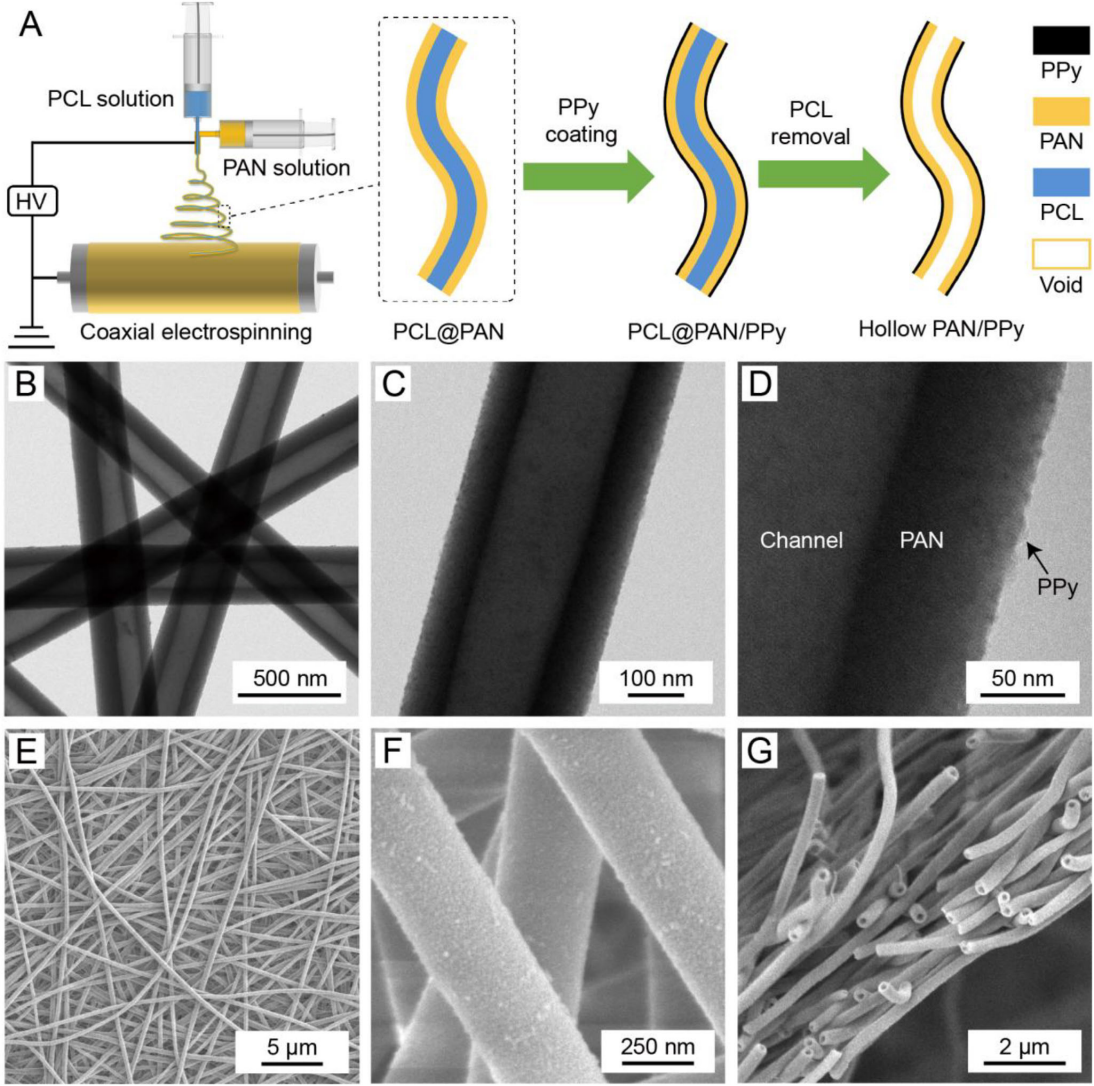
(A) Schematic illustration of the fabrication process. (B‐D) TEM images of the PAN/PPy hollow nanofibers at increasing magnifications, revealing the internal channel and the PPy coating on the PAN shell. (E) SEM image of a nonwoven mat of the PAN/PPy hollow nanofibers. (F) Magnified SEM image showing the surface of the hollow nanofibers in detail. (G) Cross‐sectional SEM image of the hollow nanofibers.

We used transmission electron microscopy (TEM) to confirm the hollow structure and the PPy coating. The low‐magnification images in Figure 1B,C indicate that the PAN/PPy nanofibers had a continuous tubular structure, whereas the magnified image in Figure 1D shows the straight channel, the PAN supporting shell, and the rough PPy coating on the outer surface. Two control samples were also examined to elucidate the origin of the rough surface layer. Specifically, the PCL@PAN/PPy fibers prior to core removal showed a continuous rough layer of PPy on the surface (Figure S3), whereas the PAN hollow fibers prepared without PPy coating showed a much smoother outer surface (Figure S4). These controls confirm that the rough outer surface mainly came from PPy deposition rather than from the PCL residue left behind during core removal.

Scanning electron microscopy (SEM) images further demonstrate that the hollowing process preserved the nonwoven network. The mat of PAN/PPy hollow nanofibers retained the porous morphology over a large area (Figure 1E), and the magnified image showed a granular surface texture arising from PPy coating (Figure 1F). As a control, the PCL@PAN core–shell nanofibers exhibited a smoother surface (Figure S5), indicating that the surface roughening mainly arose from the PPy coating. The cross‐sectional SEM image in Figure 1G clearly shows numerous open ends of the fibers, confirming successful core removal and the formation of internal channels.

### 2.2 Structural Features and Mechanical Properties of the Nanofiber Mats

After confirming the hollow morphology, we quantified the structural dimensions. As shown in Figure 2A,B, the nanofibers had an average outer diameter of 321 ± 37 nm, while their internal channels had an average diameter of 154 ± 21 nm. Both distributions approximately followed a Gaussian profile, indicating good dimensional uniformity. Assuming a cylindrical cross‐section, the channel would account for 23% of the cross‐sectional area of each fiber. As shown in Figure 2C, the density decreased from 0.20 g cm^−3^ for the solid samples to 0.16 g cm^−3^ for the hollow samples, corresponding to a 20% reduction. The calculated porosity increased from 83.0% for the solid mat to 86.5% for the hollow mat. We then used Fourier transform infrared spectroscopy (FTIR) to verify the removal of PCL. As detailed in Figure 2D, for the PCL@PAN/PPy sample, the nitrile band of PAN appeared at 2240 cm^−1^ [25], together with a set of characteristic ester bands of PCL, including the C = O stretching band at 1725 cm^−1^ and the C–O/C–O–C stretching bands at 1295, 1245, and 1190 cm^−1^ [26]. After treatment with chloroform, the nitrile band of PAN remained clearly visible, whereas the PCL‐related ester bands were reduced to near the baseline. The bands of PPy were not clearly resolved because they mainly overlap with the bands of PAN in the fingerprint region [27]. This result indicates effective removal of the PCL core while preserving the PAN shell.

**FIGURE 2:**
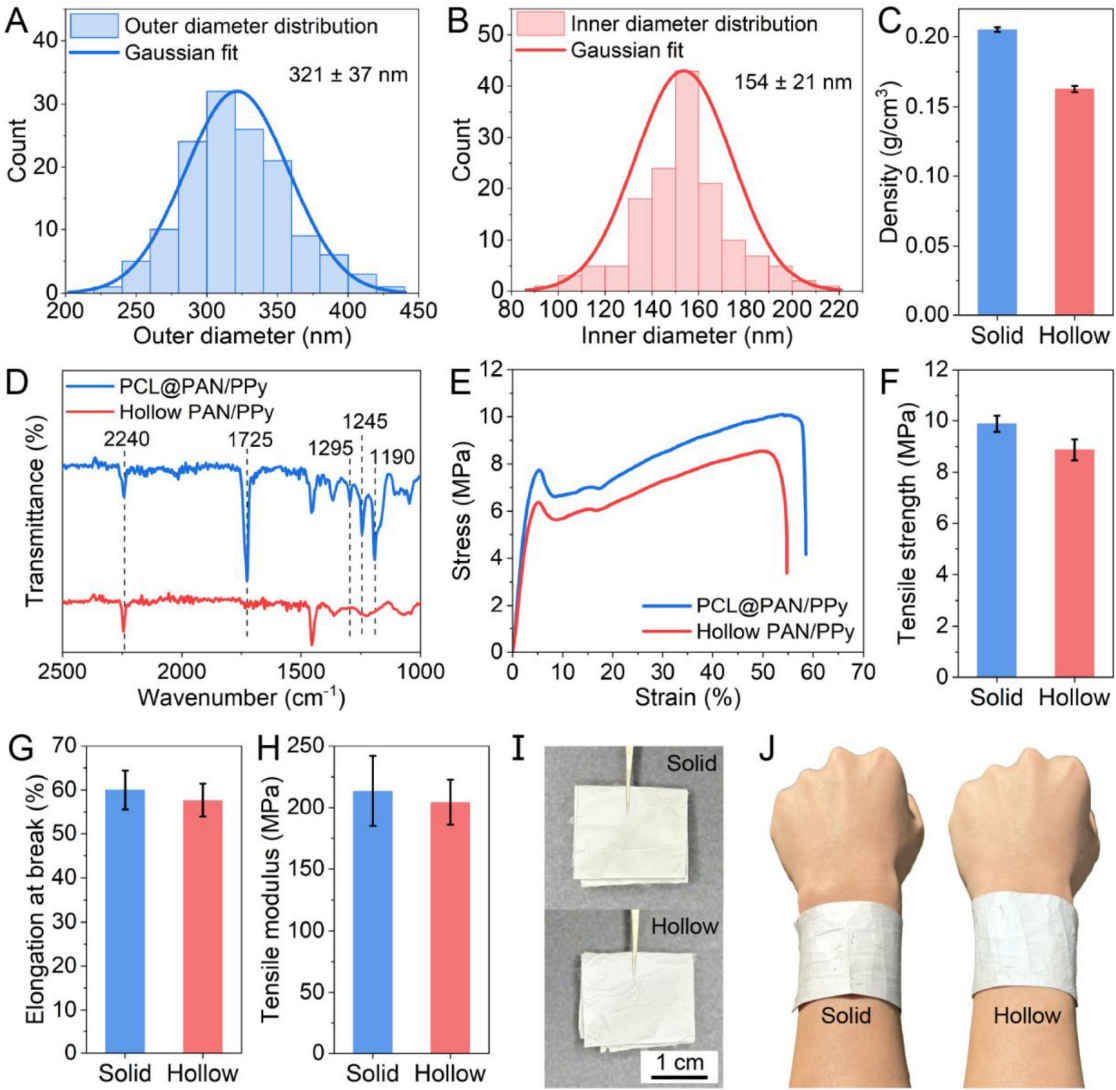
Distributions of (A) outer and (B) inner diameters of the PAN/PPy hollow nanofibers, together with Gaussian fitting. (C) Measured densities of the paired mats. (D) FTIR spectra of the paired mats of solid and hollow fibers. (E) Representative stress–strain curves, (F) tensile strength, (G) elongation at break, and (H) tensile modulus of the mats of solid and hollow nanofibers. Photographs showing paired mats of solid and hollow nanofibers (I) after repeated folding under indoor lighting, and (J) attached to the wrist under outdoor sunlight.

Tensile tests were used to compare the mechanical properties of mats of the solid and hollow nanofibers. Representative stress–strain curves indicate that both samples sustained large tensile deformation and fractured after exceeding 50% strain (Figure 2E). The tensile strength decreased from 9.9 MPa for the solid sample to 8.9 MPa for the hollow sample (Figure 2F), while the elongation at break and the tensile modulus only decreased by 3.9% and 4.2%, respectively (Figure 2G,H). Because core removal also reduced the mat density by 20%, the specific strength instead increased by 12%. Macroscopic handling tests further confirmed the retained flexibility. The mat of hollow nanofibers maintained an intact form after repeated folding, showing no visible cracking (Figure 2I). Both the solid and hollow samples could also be conformally attached to the wrist surface (Figure 2J) and rolled around glass rods (Figure S6).

### 2.3 Brighter Appearance due to Enhanced Visible Backscattering

We next examined how the internal channels affected the optical response of the mats. As shown in Figure 3A, both samples appeared light gray on white clothing, but the hollow sample was visibly brighter than the solid counterpart under outdoor shade and direct sunlight. The diffuse reflectance spectra in Figure 3B confirmed this visual difference. In the visible region, the hollow sample showed higher diffuse reflectance than the solid sample, with a larger difference around 400–600 nm that gradually narrowed toward 800 nm. After weighting the spectra with the photopic response of the human eye, the diffuse reflectance was found to increase from 59.7% for the solid mat to 64.7% for the hollow sample (Figure S7). To clarify the optical effect of the internal channels, we simulated the scattering response of individual fibers with matched dimensions. The hollow fiber exhibited stronger backscattering efficiency than the solid counterpart in the visible region, but lower backscattering in the near‐infrared region (Figure 3C; Figure S8A). The fraction of backscattering showed a similar trend (Figure S8B). The angular distributions of light scattering (Figure 3D; Figure S9) further indicate that the hollow nanofiber redirected more visible light into the backward angular range of 90°–270°.

**FIGURE 3:**
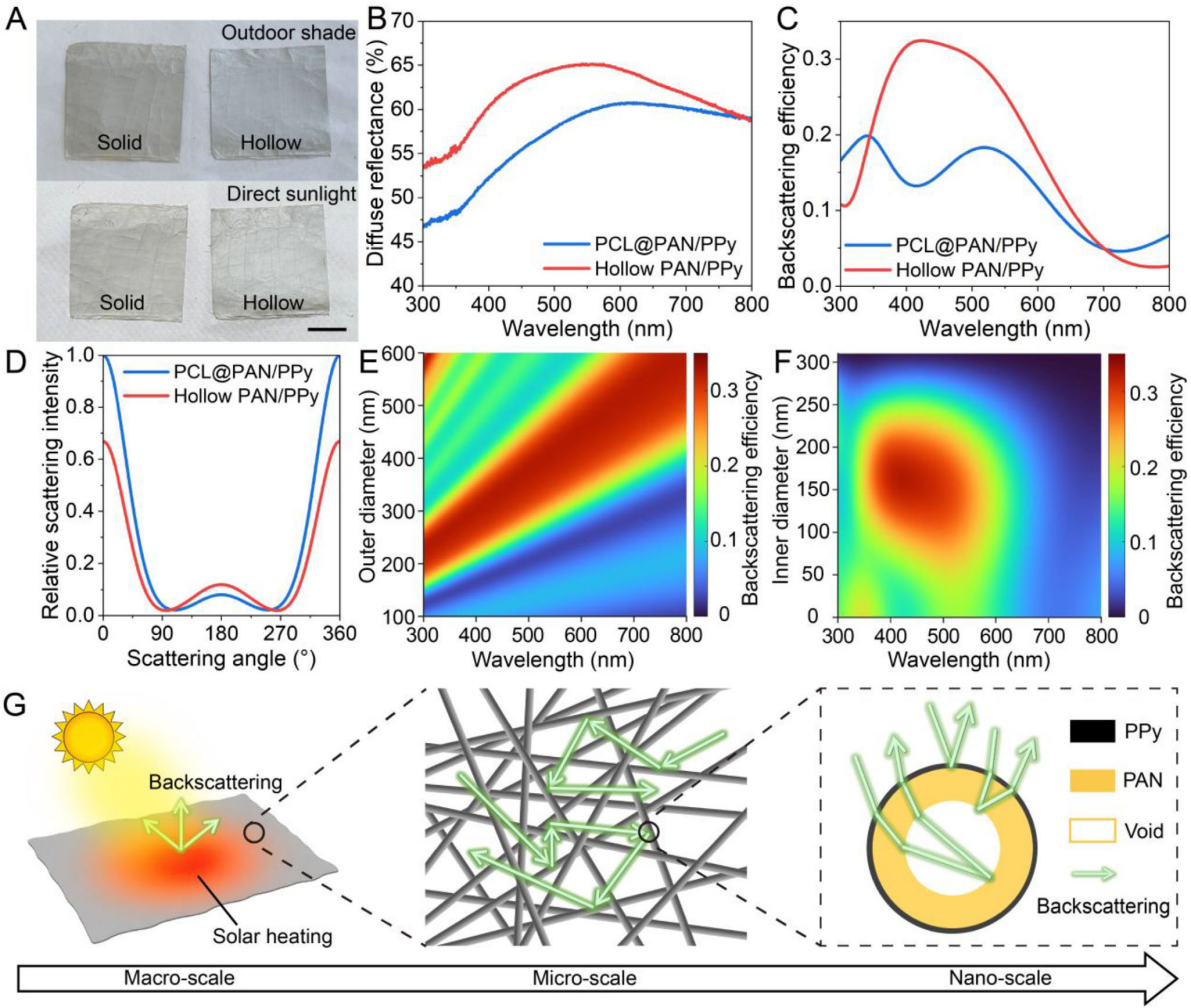
(A) Photographs of paired mats of solid and hollow nanofibers under outdoor shade and direct sunlight. Scale bar: 2 cm. (B) Diffuse reflectance spectra of paired mats of solid and hollow nanofibers, averaged from three pieces of each sample type. (C) Simulated wavelength‐dependent backscattering efficiency of the solid and hollow nanofibers. (D) Simulated angular scattering distributions of the solid and hollow nanofibers at a wavelength of 550 nm. The scattering intensity was normalized to the maximum intensity between the solid and hollow curves. (E) Two‐dimensional map of the backscattering efficiency of a PAN/PPy hollow nanofiber as a function of wavelength and outer diameter, with the inner‐to‐outer diameter ratio fixed at 0.5. (F) Two‐dimensional map of the backscattering efficiency of a PAN/PPy hollow nanofiber as a function of wavelength and inner diameter, at a fixed outer diameter of 320 nm. (G) Schematic illustration of backscattering from the fiber network and cross‐section of a hollow nanofiber.

We then performed two‐dimensional parameter scans to determine whether this enhancement depends on the hollow structure and inner/outer fiber diameter. When the ratio of inner‐to‐outer diameters was fixed at 0.5, the map showed that fibers with outer diameters in the range of 300–400 nm produced strong backscattering across the visible region (Figure 3E). This effective range of diameters includes the experimentally measured outer diameter of about 320 nm. In comparison, the solid fibers showed weaker and more discrete scattering regions in the same scan (Figure S10). This result indicates that the hollow structure of proper dimensions can enhance the backscattering of visible light by modifying the scattering mode through the additional air/polymer interface. The role of channel size was further examined by fixing the outer diameter at 320 nm while varying the inner diameter (Figure 3F). The efficiency of light backscattering increased with channel size, reaching a maximum near 160 nm and then decreasing. The measured inner diameter of about 154 nm was close to this optimal size. Smaller channels contribute more weakly because the cavity becomes too small relative to the wavelength to scatter visible light effectively. Increasing the channel size enhances cavity‐induced scattering, but excessive enlargement would thin the PAN/PPy shell, reducing the effective scattering volume. As a result, the efficiency of backscattering reaches a maximum at an intermediate channel size.

Figure 3G summarizes how the hollow structure produces a brighter appearance. The incident light is repeatedly scattered by the randomly oriented fibers in the nonwoven mat. Within each hollow fiber, the outer air/PPy interface and the inner PAN/cavity interface act as two scattering interfaces separated by a shell of approximately 80 nm in thickness. As a result, more visible light is redirected into the backward hemisphere at the single‐fiber level, and repeated scattering within the mat accumulates this backward component into stronger diffuse reflectance [28, 29]. We further simulated the scattering response of a mat consisting of randomly positioned nanofibers, with the repeated scattering between fibers included (Figure S11). The hollow mat still showed stronger backscattering than the solid counterpart in the range of 400–600 nm, indicating that the enhanced backscattering of individual hollow fibers was retained in the mat.

### 2.4 Higher Solar‐Heating Temperature Through Heat Retention

We measured the solar‐heating response under 1 sun irradiation using a solar simulator. As shown by the infrared thermal images in Figure 4A,B, the hollow sample reached a higher surface temperature than the solid counterpart. Starting from 17°C, the solid sample reached about 42°C, corresponding to a temperature rise of 25°C. In comparison, the hollow sample was heated to 45°C, with a temperature rise of 28°C, showing a 12% greater temperature rise. Repeated light‐on/off cycles confirmed that this temperature difference was consistent rather than a single‐measurement fluctuation (Figure 4C). During three consecutive cycles, the heating curve of the hollow sample remained above that of the solid sample, while both samples cooled back to the same ambient temperature after the light was turned off. The cycle‐averaged temperature difference between the hollow and solid samples increased rapidly within the first 200 s and then gradually reached a plateau region after 800 s (Figure S12).

**FIGURE 4:**
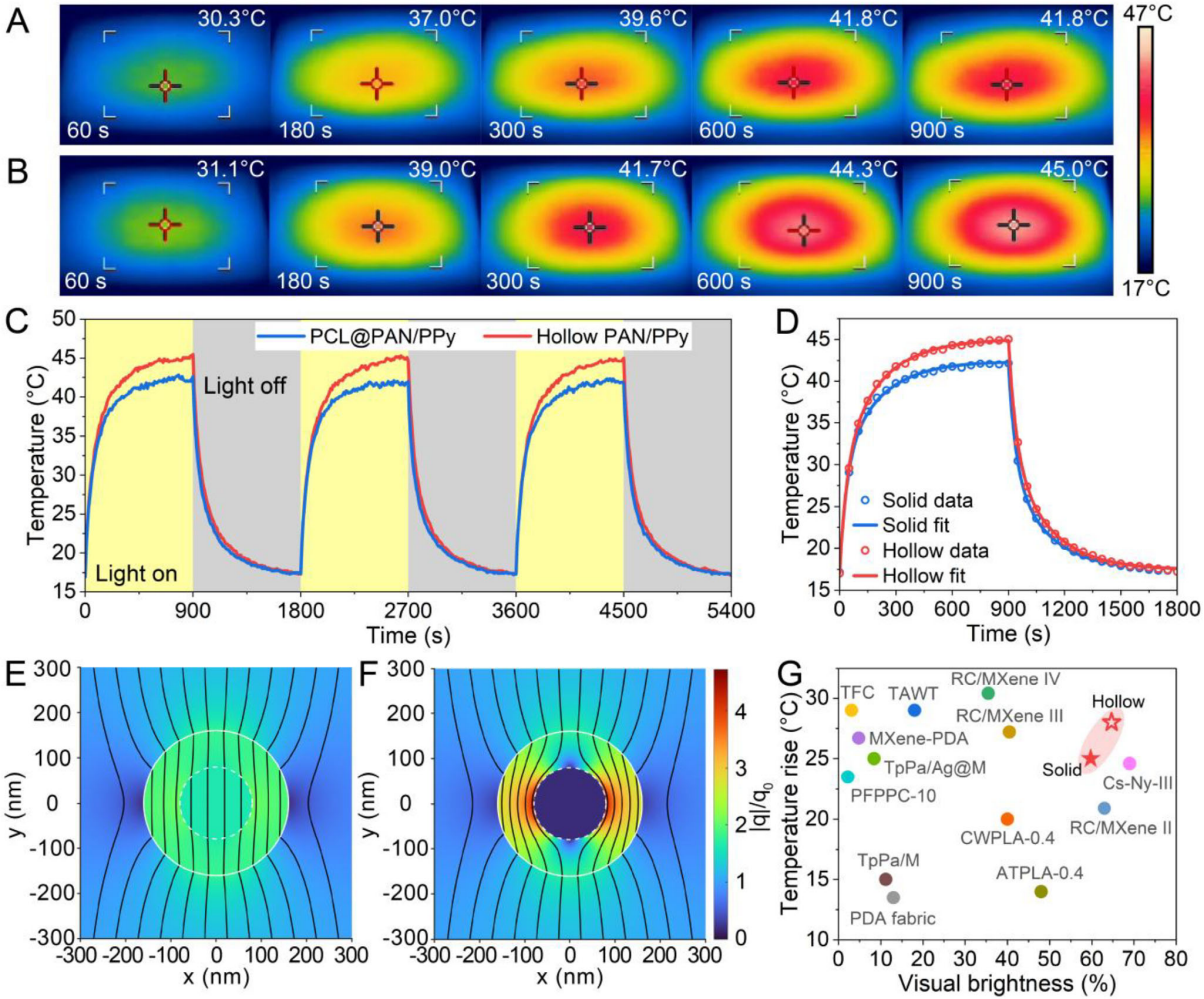
Infrared thermal images of paired mats of (A) solid and (B) hollow nanofibers under 1 sun irradiation from a solar simulator. The labeled temperature is the crosshair‐tracked maximum temperature. (C) Temperature–time profiles of the paired mats during repeated light on and off cycles. (D) Cycle‐averaged experimental temperature data and corresponding fits using the substrate‐coupled thermal model for the heating and cooling processes. Simulated heat flux distributions in a cross‐section of (E) solid and (F) hollow nanofibers under a vertical temperature gradient, with black streamlines showing the heat flow paths. (G) Literature comparison of visual brightness and temperature rise for reported solar‐heating textiles and this work. The filled and open red star markers represent the solid and hollow samples in this work, respectively.

To identify the origin of this higher temperature rise, we fitted the cycle‐averaged heating and cooling profiles using thermal models. The conventional single‐exponential model [30] captured the overall trend but gave relatively large fitting errors (Figure S13; Table S1). Because the sample was placed on a substrate during the test, we introduced a substrate‐coupled model that separates the sample surface response from the slower thermal response of the underlying support. This model substantially improved the fitting quality (Figure 4D). As listed in Table S2, the fitted parameters indicate that the hollow sample had an 8.3% lower effective heat‐loss rate, while its initial heating rate was only 1.2% higher than that of the solid counterpart. This contrast suggested that the higher solar‐heating temperature of the hollow sample mainly arose from reduced heat dissipation rather than increased heat generation. Since the paired samples were measured under the same illumination, substrate, and ambient conditions, the external heat‐transfer conditions were comparable. Thus, the lower effective heat‐loss rate is associated with suppressed heat conduction within the fibrous mat.

We then simulated two‐dimensional steady‐state heat conduction across a single fiber under a uniform temperature gradient (Figure 4E,F). In the solid fiber, the PCL core carried about 45% of the heat flow across the fiber. After core removal, only 5% of the heat flow passed through the air channel (Figure S14A), and the streamlines bypassed the channel and crowded into the surrounding shell. Because the channel diameter is comparable to the mean free path of air, the thermal conductivity of the confined air was less than half the bulk value. The thermal conductivity across the fiber therefore decreased from 0.24 to 0.16 W m^−1^ K^−1^, a reduction of 33% (Figure S14B,C). Figure S15 summarizes this heat‐retention mechanism. For solid fibers, the PCL core provides a continuous polymer pathway for heat transfer across the fiber's interior and through the fibrous network. After core removal, the channel disrupts heat propagation and increases the resistance to heat transfer across individual fibers. As a result, the hollow sample retains heat more effectively and reaches a higher solar‐heating temperature [19, 31].

We then compared the solid and hollow samples in this work with reported solar‐heating textiles [14, 32, 33, 34, 35, 36, 37, 38] in terms of visual brightness and temperature rise (Figure 4G; Table S3). Most reported textiles with large temperature rises typically show low visual brightness because their solar‐heating performance is mainly achieved through strong solar absorption. In contrast, hollowing out the nanofibers enabled the sample to achieve enhanced visual brightness and higher temperature rise than its paired solid control, demonstrating a general structural strategy to overcome this trade‐off.

### 2.5 Side‐by‐Side Solar‐Heating Comparison Under Indoor and Outdoor Conditions

We also conducted side‐by‐side tests to verify the advantage of the hollow sample under identical testing conditions. Unlike the separate measurements shown in Figure 4, the paired mats of solid and hollow nanofibers were placed in the same illumination field, on the same substrate, and within the same infrared imaging frame (Figure S16). A small gap was kept between the two samples to reduce lateral heat transfer while separating the two temperature regions in the infrared images (Figure 5A). Indoor co‐irradiation showed that the maximum‐temperature region remained on the hollow sample, confirming its higher solar‐heating temperature under the same illumination and substrate conditions (Figure 5B; Movie S1). The absolute temperatures were slightly lower than those in the separate test because the two mats shared the same solar‐simulator beam.

**FIGURE 5:**
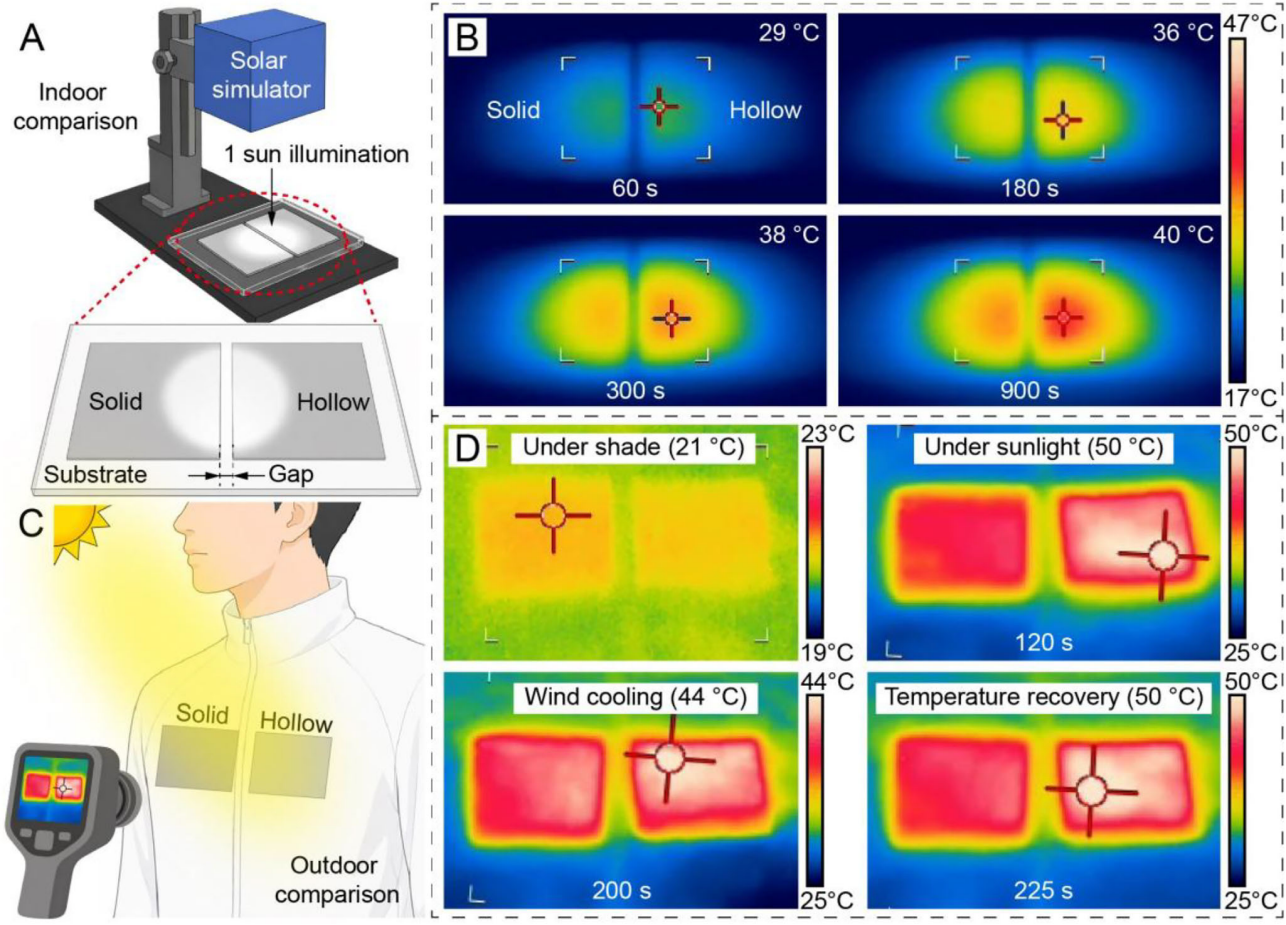
(A) Schematic illustration of the indoor side‐by‐side comparison under the solar simulator with 1 sun illumination, where paired mats of solid and hollow nanofibers were placed on the same substrate with a gap between the mats. (B) Representative infrared thermal images of the paired samples from the indoor co‐irradiation test at selected heating times. The labeled temperature is the crosshair‐tracked maximum temperature. (C) Schematic illustration of the comparison of outdoor wearable solar heating, with paired mats of solid and hollow nanofibers attached side by side on clothing. (D) Representative infrared thermal images from the outdoor side‐by‐side test. The labeled temperature is the crosshair‐tracked maximum temperature.

The side‐by‐side comparison was then extended to outdoor sunlight. The paired mats were attached to white clothing to compare their response under real sunlight, ambient wind, and a curved wearable surface (Figure 5C). Both samples remained close to ambient temperature (21°C) under shade, and the tracked maximum‐temperature point was initially located on the solid sample, showing that the hollow sample did not start at a higher temperature. Upon direct sunlight exposure, the maximum‐temperature region shifted to the hollow sample and quickly reached 50°C at *t* = 120 s, corresponding to a temperature rise of about 29°C (Figure 5D; Movie S2). Because of wind cooling, the tracked temperature decreased to 44°C, but the hollow sample remained hotter than the solid counterpart. When the wind stopped, the temperature recovered to 50°C at *t* = 225 s, and the hollow sample remained at a higher temperature. These results demonstrate the robust solar‐heating advantage of the hollow sample under outdoor conditions. A similar trend was also observed for the nanofiber mats supported on a polystyrene foam (Figure S17), further confirming the reproducibility of this solar‐heating material regardless of the substrate.

## 3 Conclusion

In summary, we have demonstrated that a nonwoven mat of PAN/PPy hollow nanofibers can be brighter yet hotter than its paired solid counterpart during solar heating. With suitable hollow‐fiber dimensions, the mat enhanced visible backscattering and diffuse reflectance, making it visibly brighter. The hollow structure also reduced heat dissipation through the fibrous network, enabling the hollow mat to achieve a 12% higher temperature rise than the paired solid mat under 1 sun irradiation. Indoor and outdoor side‐by‐side comparisons further confirmed that the mat of hollow nanofibers consistently remained warmer. These results identify hollowing nanofibers as a design principle to reduce heat dissipation while enhancing light scattering in solar‐heating textiles.

## Author Contributions

**Minghong Wu**: conceptualization, methodology, investigation, validation, visualization, writing – original draft, data curation, writing – review and editing. **Yuxuan Meng**: investigation. **Huajian Ji**: investigation, visualization. **Jianlong He**: investigation, writing – review and editing. **Min Hao**: investigation, writing – review and editing. **Younan Xia**: conceptualization, writing – review and editing, supervision, resources, project administration, funding acquisition.

## Conflicts of Interest

The authors declare no conflicts of interest.

## Supporting information




**Supporting File 1**: smll76477‐sup‐0001‐MovieS1.mp4.

**Supporting File 2**: smll76477‐sup‐0002‐MovieS2.mp4.

**Supporting File 3**: smll76477‐sup‐0003‐SuppMat.pdf.

## Data Availability

The data that support the findings of this study are available from the corresponding author upon reasonable request.
