# Supplementary material for: Hollowing Out Nanofibers for Enhanced Visual Brightness and More Effective Solar Heating
Source: Small. 2026 Oct 27;22(63):e76477. doi: 10.1002/smll.76477 (PMC13755988; doi:10.1002/smll.76477)
Supplement: Supplementary file 1 [file SMLL-22-e76477-s003.pdf]

Supporting Information

Hollowing Out Nanofibers for Enhanced Visual Brightness and More Effective Solar Heating

Minghong Wu[1], Yuxuan Meng[2], Huajian Ji[1], Jianlong He[3,4], Min Hao[3,4], and Younan Xia[1,2,3,4,5,*]

[1]School of Chemical and Biomolecular Engineering, Georgia Institute of Technology, Atlanta, Georgia 30332 (USA)

[2]School of Chemistry and Biochemistry, Georgia Institute of Technology, Atlanta, Georgia 30332 (USA)

[3]The Wallace H. Coulter Department of Biomedical Engineering, Georgia Institute of Technology and Emory University, Atlanta, Georgia 30332 (USA)

[4]Department of Materials Science and Engineering, Johns Hopkins University, Baltimore, Maryland 21218 (USA)

[5]Department of Biomedical Engineering, Johns Hopkins University, Baltimore, Maryland 21218 (USA)

*Corresponding author E-mail: yxia70@jh.edu

Experimental Section

Chemicals and materials

Polyacrylonitrile (PAN, $M_v \approx 150{,}000$), poly(ε-caprolactone) (PCL, $M_n \approx 80{,}000$), chloroform, *N,N*-dimethylformamide (DMF), pyrrole, and iron(III) chloride hexahydrate ($FeCl_3 \cdot 6H_2O$) were purchased from Sigma-Aldrich and used as received.

Preparation of mats of PCL@PAN core-shell nanofibers

Mats of PCL@PAN nanofibers were fabricated by coaxial electrospinning. A PAN solution (11 wt%) in DMF was used as the shell solution, and a PCL solution (10 wt%) in DMF was prepared at 50 °C and used as the core solution. Electrospinning was performed using a coaxial spinneret consisting of a 17-gauge outer needle and a 22-gauge inner needle. The shell and core flow rates were 0.9 and 0.3 mL h^{-1}, respectively. A voltage of 14 kV was applied with a tip-to-collector distance of 10 cm. The environmental conditions were maintained at 25 °C and 25% relative humidity (RH). The as-spun nanofiber mats were dried overnight at room temperature.

Preparation of mats of PAN/polypyrrole (PPy) hollow nanofibers

PPy was deposited on the PCL@PAN nanofibers by *in situ* polymerization. An aqueous pyrrole solution (5 mM, 200 mL) and an aqueous $FeCl_3$ solution (10 mM, 200 mL) were mixed at a 1:1 volume ratio to prepare the reaction solution. The mats of PCL@PAN nanofibers (18 cm × 6 cm × 60 μm) were immersed in the freshly prepared reaction solution at room temperature for 6 h to obtain mats of PCL@PAN/PPy nanofibers, followed by thorough rinsing with deionized water and drying overnight. For paired comparison, a mat of PCL@PAN/PPy nanofibers was cut into multiple pieces. Some pieces were retained as solid controls, whereas the remaining pieces were immersed in chloroform at room temperature to selectively remove the PCL core, yielding mats of PAN/PPy hollow nanofibers.

Characterizations

Transmission electron microscopy (TEM) was performed using a Hitachi HT7700 microscope to examine the structural features of the nanofibers. Scanning electron microscopy (SEM) was conducted on a Hitachi SU8230 field-emission SEM to analyze the mat-scale morphology, surface texture, and hollow features in cross-sectional views. Outer fiber diameters were measured from

SEM images, and inner diameters were measured from TEM images using ImageJ. About 150 fibers were measured for each diameter distribution. The density of the nanofiber mats was calculated from the measured mass and geometric volume. Fourier transform infrared (FTIR) spectra were collected using a Shimadzu IRAffinity-1 spectrometer to compare chemical composition changes before and after PCL removal. Tensile properties of the nanofiber mats were measured following ASTM D882 using an Instron 5944 universal testing system equipped with a 1 kN load cell. Diffuse reflectance spectra were recorded using an Agilent Cary 5000 spectrophotometer with an integrating sphere accessory. To quantify visual brightness, the photopic-weighted diffuse reflectance was calculated from 400 to 800 nm by weighting the measured diffuse reflectance spectrum with the CIE photopic luminous efficiency function, $R_{\text{photopic}} = \sum R(\lambda)V(\lambda)/\sum V(\lambda)$, where $R(\lambda)$ is the diffuse reflectance and $V(\lambda)$ is the photopic weighting function. Solar-heating measurements were carried out under standardized 1 sun illumination provided by an Ossila LED solar simulator. Nanofiber mats with dimensions of 6 cm × 6 cm × 60 µm were placed on a polystyrene (PS) substrate, and a FLIR E60 infrared camera was used to record the time-dependent surface temperature and temperature-field distributions. For side-by-side tests, paired mats of solid and hollow nanofibers were placed on the same substrate with a 3 mm gap and monitored within the same infrared imaging frame. Outdoor side-by-side tests were conducted at Georgia Tech, Atlanta (33.78°N, 84.40°W), from 13:00 to 14:00 on April 10, 2026, under 20 °C, 40% RH, and a wind speed of 5 km h^{-1}. The outdoor solar irradiance (967 W m^{-2}) was obtained from NASA POWER hourly data. Photographs of the samples were taken with an iPhone 15 to document their appearance, dimensions, flexibility, and wearability.

Calculation of Porosity

The porosity of the nanofiber mats was calculated from the measured density of the mat and the density of the fiber material. The densities of PCL and PAN were taken from Sigma-Aldrich product information as 1.145 and 1.184 g cm^{-3} at 25 °C, respectively. Because the PPy coating was thin, its contribution to the fiber density was neglected in this estimation.

For the solid PCL@PAN/PPy fibers, the PCL core area fraction was calculated from the measured inner and outer diameters:

$$f_{\text{core}} = \left(\frac{d_{\text{inner}}}{d_{\text{outer}}}\right)^2 = 0.23 \qquad (S1)$$

The effective density of the solid fiber was then calculated as:

$$\rho_{\text{solid fiber}} = f_{\text{core}}\rho_{\text{PCL}} + (1 - f_{\text{core}})\rho_{\text{PAN}} = 1.175 \text{ g cm}^{-3} \tag{S2}$$

The porosity of the solid mat was calculated as:

$$\varepsilon_{\text{solid}} = \left(1 - \frac{\rho_{\text{solid mat}}}{\rho_{\text{solid fiber}}}\right) \times 100\% = 83.0\% \tag{S3}$$

For the hollow mat, the internal channel was included as void volume. The total porosity was calculated as:

$$\varepsilon_{\text{hollow}} = \left(1 - \frac{\rho_{\text{hollow mat}}}{\rho_{\text{PAN}}}\right) \times 100\% = 86.5\% \tag{S4}$$

Theory and Modeling

All calculations were performed in MATLAB.

Light scattering model of single fibers

Light scattering was calculated using a cylindrical electromagnetic scattering model for an infinitely long coaxial fiber in air. The fiber was simplified as a two-layer cylinder consisting of a PAN/PPy shell and an inner core. For the PCL@PAN/PPy fiber, the inner core was PCL; for the hollow PAN/PPy fiber, the inner core was an air cavity. In the fixed-geometry model, the outer fiber diameter was set to 320 nm, and the inner core or cavity diameter was set to 160 nm. The refractive indices of air, PCL core, cavity, and PAN/PPy shell were set as $m_{\text{air}} = 1.00 + 0i$, $m_{\text{core}} = 1.46 + 0i$, $m_{\text{cavity}} = 1.00 + 0i$, and $m_{\text{shell}} = 1.52 + 0.003i$, respectively, where the shell extinction coefficient was obtained by matching the simulated diffuse reflectance of the solid mat to the measured value.

For each wavelength, the size parameter was calculated as

$$x = \frac{2\pi R_{\text{out}}}{\lambda} \tag{S5}$$

where R_{out} is the outer radius of the fiber and λ is the incident wavelength. The electromagnetic field was expanded using cylindrical harmonics, and the scattering coefficients were obtained by applying the continuity conditions at the air/shell and shell/core interfaces. Both transverse magnetic (TM) and transverse electric (TE) polarizations were calculated and averaged to approximate the scattering response under unpolarized incident light.

The total scattering efficiency Q_{sca} was calculated from the cylindrical scattering coefficients as

$$Q_{\mathrm{sca}} = \frac{2}{x}\sum_{n=0}^{n_{\max}} w_n \mid a_n \mid^2 \quad (S6)$$

where a_n is the scattering coefficient of the n-th cylindrical harmonic order, w_n is the weighting factor of the expansion term, x is the size parameter, and $n_{\max}$ is the maximum expansion order used to truncate the cylindrical harmonic series.

The angular scattering intensity for unpolarized light was calculated as

$$I(\phi) = \frac{1}{2}(\mid S_{\mathrm{TM}}(\phi) \mid^2 + \mid S_{\mathrm{TE}}(\phi) \mid^2) \quad (S7)$$

where $S_{\mathrm{TM}}(\phi)$ and $S_{\mathrm{TE}}(\phi)$ are the scattering amplitude functions under TM and TE polarizations, respectively, and ϕ is the scattering angle.

The backscattering fraction was defined as

$$f_{\mathrm{back}} = \frac{\int_{\pi/2}^{3\pi/2} I(\phi)\, d\phi}{\int_0^{2\pi} I(\phi)\, d\phi} \quad (S8)$$

where the backward angular range corresponds to $\pi/2 \leq \phi \leq 3\pi/2$. The backscattering efficiency was then calculated as

$$Q_{\mathrm{back}} = f_{\mathrm{back}} Q_{\mathrm{sca}} \quad (S9)$$

Two parameter sweeps were performed: (1) the outer fiber diameter was varied from 100 to 600 nm while maintaining an inner-to-outer diameter ratio of 0.5; (2) the hollow cavity diameter was varied from 0 to 310 nm at a fixed outer fiber diameter of 320 nm.

Light scattering model of nanofiber mats

Light scattering of the mat was calculated using a multiple-fiber electromagnetic scattering model. The mat was simplified as a rectangular region with a width of 10 μm and a thickness of 2.5 μm, in which 53 fibers were randomly placed. The number of fibers was determined from the porosity of 83.0% for the solid mat. Each fiber had the same structure and refractive indices as in the light scattering model of single fibers. The same fiber positions were used for the solid and hollow mats. A plane wave was incident normal to the mat surface. The scattered field of each fiber was expanded using cylindrical harmonics with the same scattering coefficients as in the light scattering model of single fibers. The field incident on each fiber included the incident plane wave and the fields scattered by all other fibers. The scattered fields of all fibers were solved

simultaneously, so that the repeated scattering between fibers was included. Both TM and TE polarizations were calculated and averaged.

The angular scattering intensity $I(\phi)$ of the mat was calculated from the total scattered field using Equation S7. The backscattering efficiency of the mat was calculated as

$$Q_{\text{back,mat}} = \frac{2}{\pi k W} \int_{\pi/2}^{3\pi/2} I\,(\phi)\,d\phi \tag{S10}$$

where $k = 2\pi/\lambda$ is the wavenumber and W is the width of the mat. The calculation was performed from 300 to 800 nm. The fiber positions were randomly generated 100 times, and $Q_{\text{back,mat}}$ was averaged.

Single-exponential thermal model

A single-exponential thermal model was used to fit the light-on heating and light-off cooling stages. In this model, the sample is represented by one effective temperature response.

The energy balance for the full heating–cooling cycle is written as

$$C\frac{dT_s}{dt} = Q(t) - K_s(T_s - T_\infty) \tag{S11}$$

where $T_s(t)$ is the surface temperature of the sample, T_∞ is the ambient temperature, C is the effective heat capacity of the sample, K_s is the effective heat-loss coefficient from the measured sample surface. $Q(t)$ is the effective heat-input term. During irradiation, $Q(t) = Q_0$; after the light is turned off, $Q(t) = 0$.

When irradiation starts at $t = 0$ and the initial temperature is $T_s(0) = T_\infty$, the heating-stage temperature response is

$$T_s(t) = T_\infty + \frac{Q_0}{K_s}\left(1 - e^{-\frac{K_s}{C}t}\right) \tag{S12}$$

After the light is turned off, if the light-off time is t_{off} and the temperature rise at that moment is $\Delta T_{\text{off}} = T_s(t_{\text{off}}) - T_\infty$, the cooling-stage temperature response is

$$T_s(t) = T_\infty + \Delta T_{\text{off}} e^{-\frac{K_s}{C}(t - t_{\text{off}})} \tag{S13}$$

Substrate-coupled thermal model

For nanofiber mats on a substrate, a substrate-coupled thermal model was used to account for heat exchange with the underlying support. The model contains two effective temperature responses: the sample surface temperature $T_s(t)$, corresponding to the infrared-camera measurement, and the substrate temperature $T_p(t)$, describing the slower thermal response of the PS substrate.

The energy balances for the full heating–cooling cycle are written as

$$C_s \frac{dT_s}{dt} = Q(t) - K_s(T_s - T_\infty) - K_{sp}(T_s - T_p) \quad (S14)$$

$$C_p \frac{dT_p}{dt} = K_{sp}(T_s - T_p) - K_p(T_p - T_\infty) \quad (S15)$$

where C_s and C_p are the effective heat capacities of the sample and substrate, respectively. K_s is the effective heat-loss coefficient from the sample surface, K_{sp} is the effective heat-exchange coefficient across the sample–substrate interface, and K_p is the effective heat-loss coefficient from the substrate to the surroundings. $Q(t)$ is the effective heat-input term. During irradiation, $Q(t) = Q_0$; after the light is turned off, $Q(t) = 0$.

The sample surface temperature can be expressed as

$$T_s(t) = T_\infty + \Delta T_{s,ss} + A_1 e^{\lambda_1 t} + A_2 e^{\lambda_2 t} \quad (S16)$$

where $\Delta T_{s,ss}$ is the steady-state surface temperature rise for the corresponding illumination condition. A_1 and A_2 are constants determined by the initial conditions of that time interval, and λ_1 and λ_2 are the two negative characteristic roots of the coupled thermal system.

Fiber heat-conduction model

Two-dimensional steady-state heat conduction across the fiber was calculated using a finite-volume method (FVM). The fiber was simplified as a two-layer cylinder consisting of a PAN/PPy shell and an inner core/channel, with the cross-section treated as a plane perpendicular to the fiber axis. The thermal conductivities of the PAN/PPy shell, PCL core, and surrounding air were set as 0.26, 0.20, and 0.0263 W m^{-1} K^{-1}, respectively.

The temperature field was obtained by solving the steady-state heat conduction equation

$$\nabla \cdot (k \nabla T) = 0 \quad (S17)$$

where T is the temperature and k is the thermal conductivity. A uniform temperature gradient G was applied far from the fiber. The equation was discretized on a uniform grid, with the conductivity at each cell face taken as the harmonic mean of the two adjacent cells. The temperatures on the four boundaries of the computational window were assigned from the analytical solution for a two-layer cylinder in a uniform far-field gradient, so that the finite window is equivalent to an infinite surrounding medium.

Because the cavity diameter is comparable to the mean free path of air, the conductivity of the air cavity was evaluated with the Knudsen formula as

$$k_{\text{cavity}} = \frac{k_{\text{air}}}{1 + 2\beta \text{Kn}}, \qquad \text{Kn} = \frac{\Lambda}{d} \tag{S18}$$

where Kn is the Knudsen number, Λ = 68 nm is the mean free path of air at 25 °C and 1 atm, d is the inner channel diameter, and β = 1.643 is a coefficient determined by the thermal accommodation coefficient, the specific heat ratio, and the Prandtl number of air. At d = 160 nm, Kn = 0.42 and k_{cavity} = 0.011 W m^{-1} K^{-1}.

The heat flux was obtained from the temperature field as

$$\mathbf{q} = -k\nabla T \tag{S19}$$

and its magnitude was normalized to $q_0 = k_{\text{air}}G$, the flux carried by pure air under the same gradient.

The heat flow through the fiber was obtained by integrating the flux component along the gradient direction over the fiber mid-plane, which passes through the fiber center perpendicular to the gradient:

$$F_{\text{total}} = \int_{-R_{\text{out}}}^{R_{\text{out}}} q_{\perp}\, \mathrm{d}s, \qquad F_{\text{core}} = \int_{-R_{\text{in}}}^{R_{\text{in}}} q_{\perp}\, \mathrm{d}s, \qquad F_{\text{shell}} = F_{\text{total}} - F_{\text{core}} \tag{S20}$$

where R_{in} and R_{out} are the inner and outer radii of the fiber, $q_{\perp}$ is the flux component along the applied gradient, and s is the coordinate along the mid-plane.

The thermal conductivity across the fiber was calculated from the analytical Hashin expression for a two-layer cylinder as

$$k_{\perp} = k_{\text{s}} \frac{(k_{\text{c}} + k_{\text{s}}) + f(k_{\text{c}} - k_{\text{s}})}{(k_{\text{c}} + k_{\text{s}}) - f(k_{\text{c}} - k_{\text{s}})} \tag{S21}$$

where k_{c} and k_{s} are the thermal conductivities of the core and the shell, and $f = (d/D)^2$ is the core area fraction, with D the outer fiber diameter.

This analytical solution was checked independently against the finite-volume results. For each case, the perturbation field $T - Gx$ was evaluated in the region far from the fiber, where x is the coordinate along the applied gradient. The dipole coefficient B was obtained by least-squares fitting, and the equivalent conductivity was recovered as

$$k_{\perp} = k_{\mathrm{air}} \frac{GR_{\mathrm{out}}^2 - B}{GR_{\mathrm{out}}^2 + B} \tag{S22}$$

The thermal conductivity across the fiber was calculated for inner diameters from 0 to 310 nm at a fixed outer diameter of 320 nm, and the finite-volume checks were performed at inner diameters of 80, 160, and 240 nm.

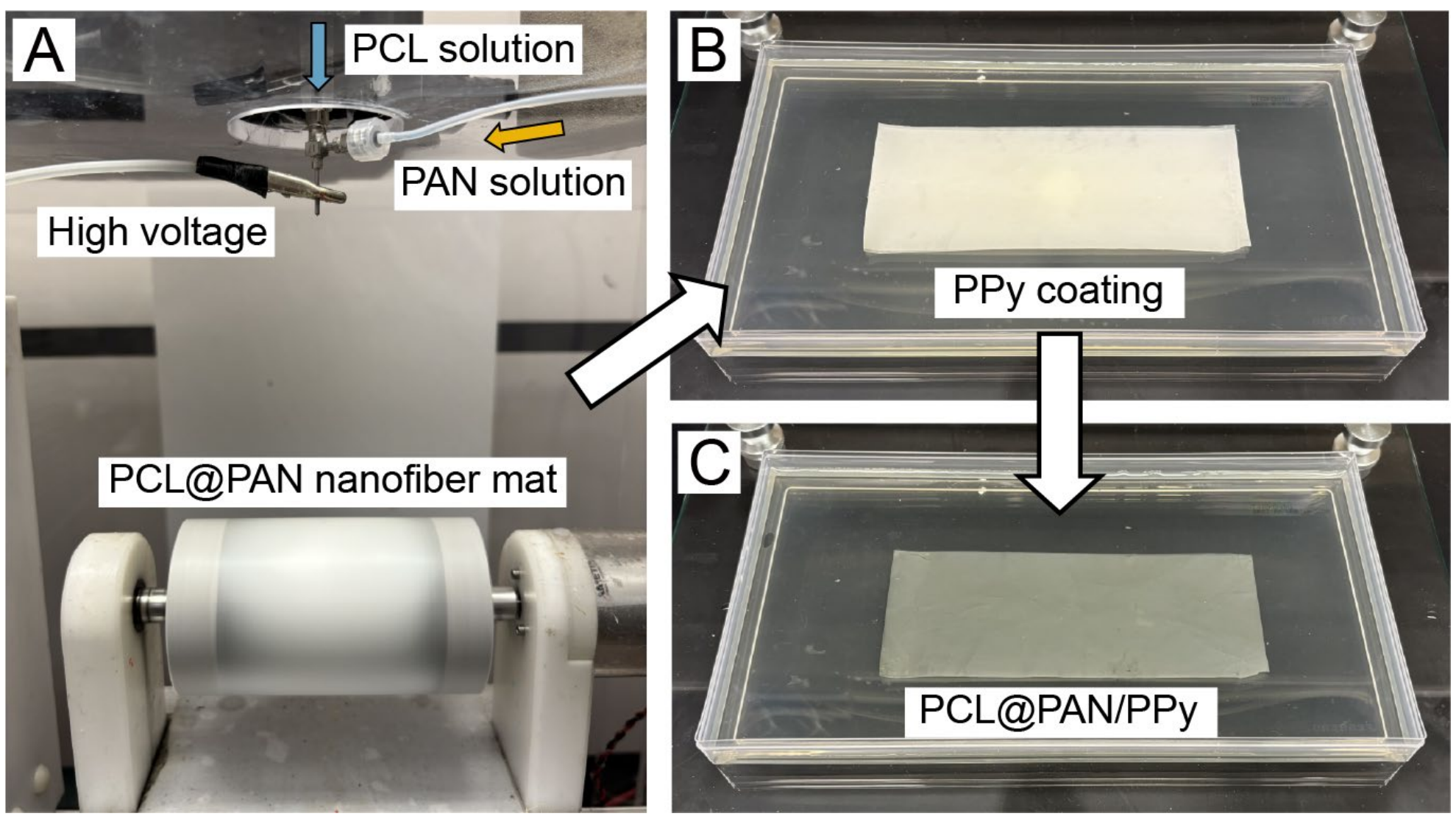

Figure S1. (A) Coaxial electrospinning setup used to prepare PCL@PAN core-shell nanofibers. (B) Mat of PCL@PAN nanofibers at the beginning of PPy coating and (C) after 6 h of PPy coating.

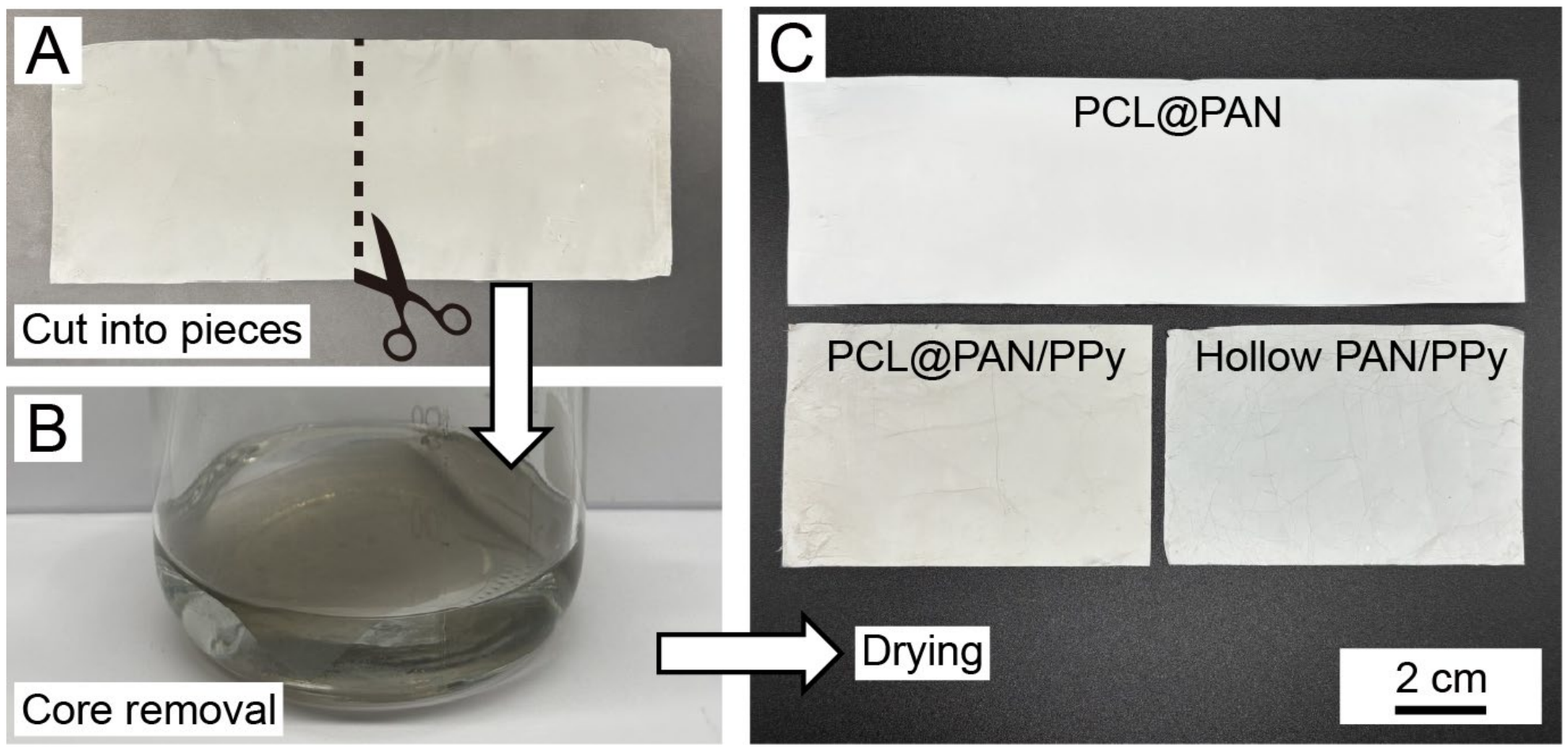

Figure S2. (A) The mat of PCL@PAN/PPy nanofibers was cut into pieces for paired comparison. (B) One half was immersed in chloroform to remove the PCL core. (C) Representative dried samples: as-spun PCL@PAN, PCL@PAN/PPy, and PAN/PPy hollow samples.

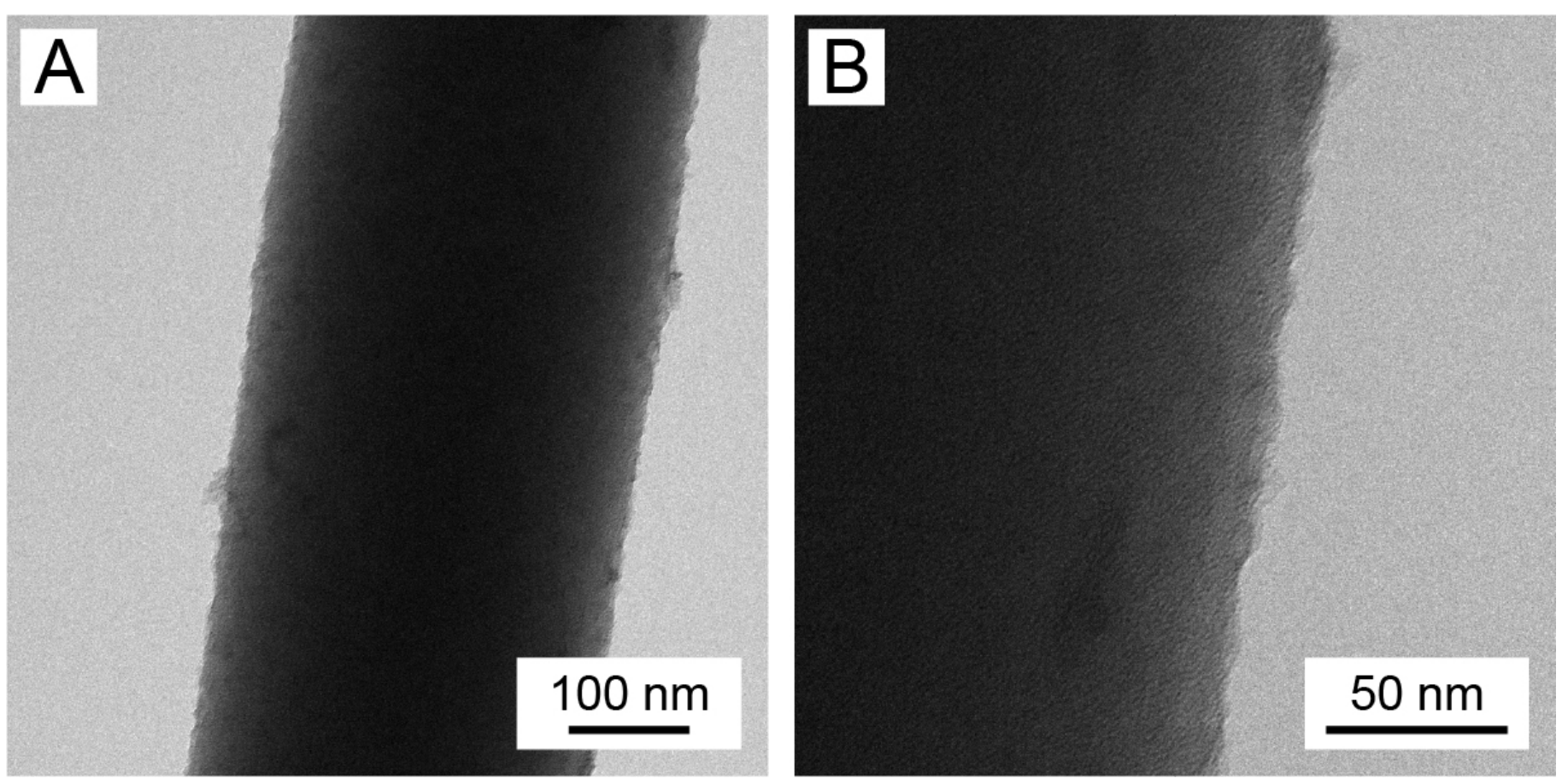

Figure S3. (A) TEM image of a PCL@PAN/PPy nanofiber and (B) magnified TEM image showing the PPy layer on the surface of the PCL@PAN nanofiber.

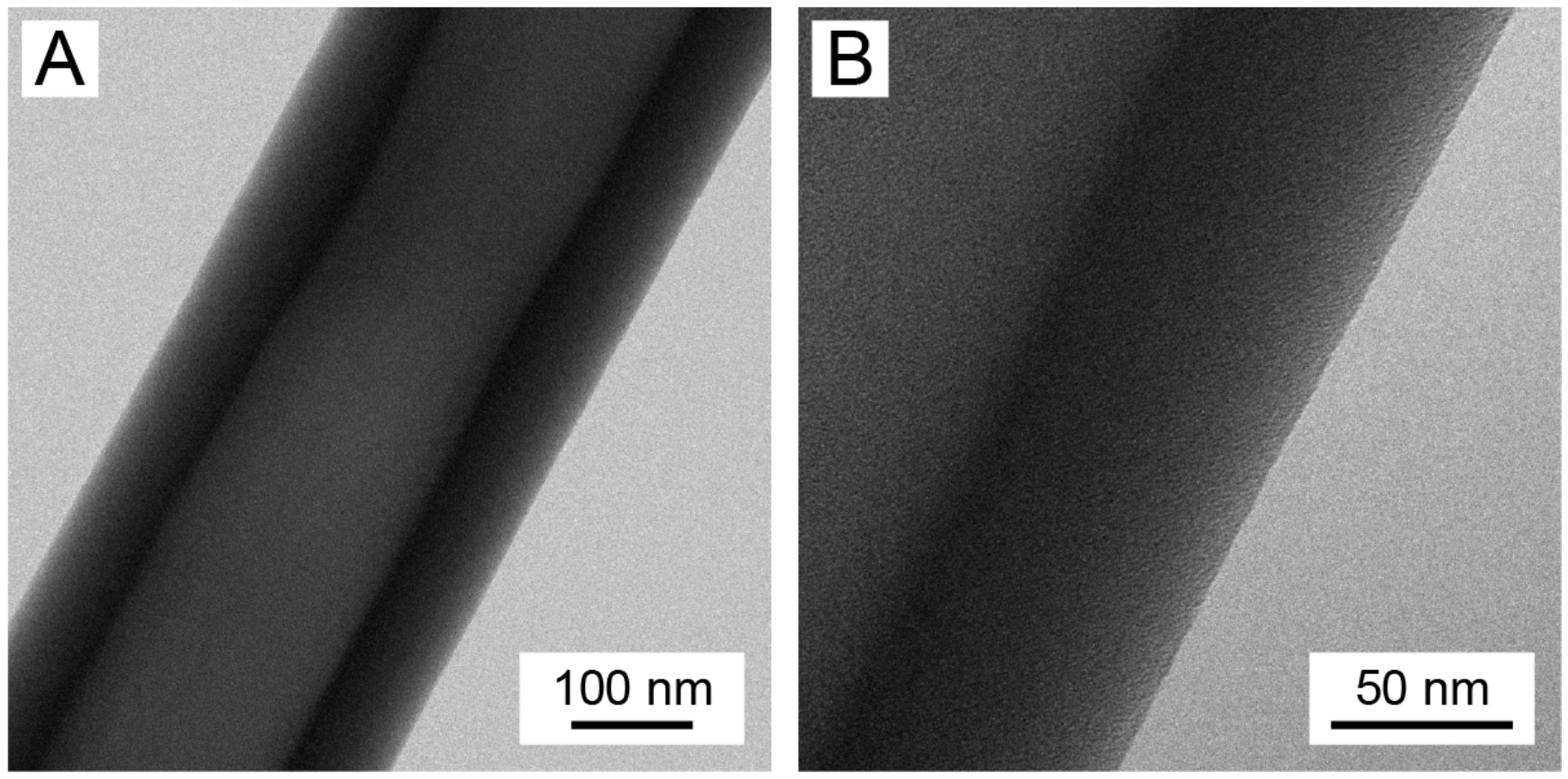

Figure S4. (A) TEM image of a PAN hollow nanofiber and (B) magnified TEM image showing its smooth outer surface.

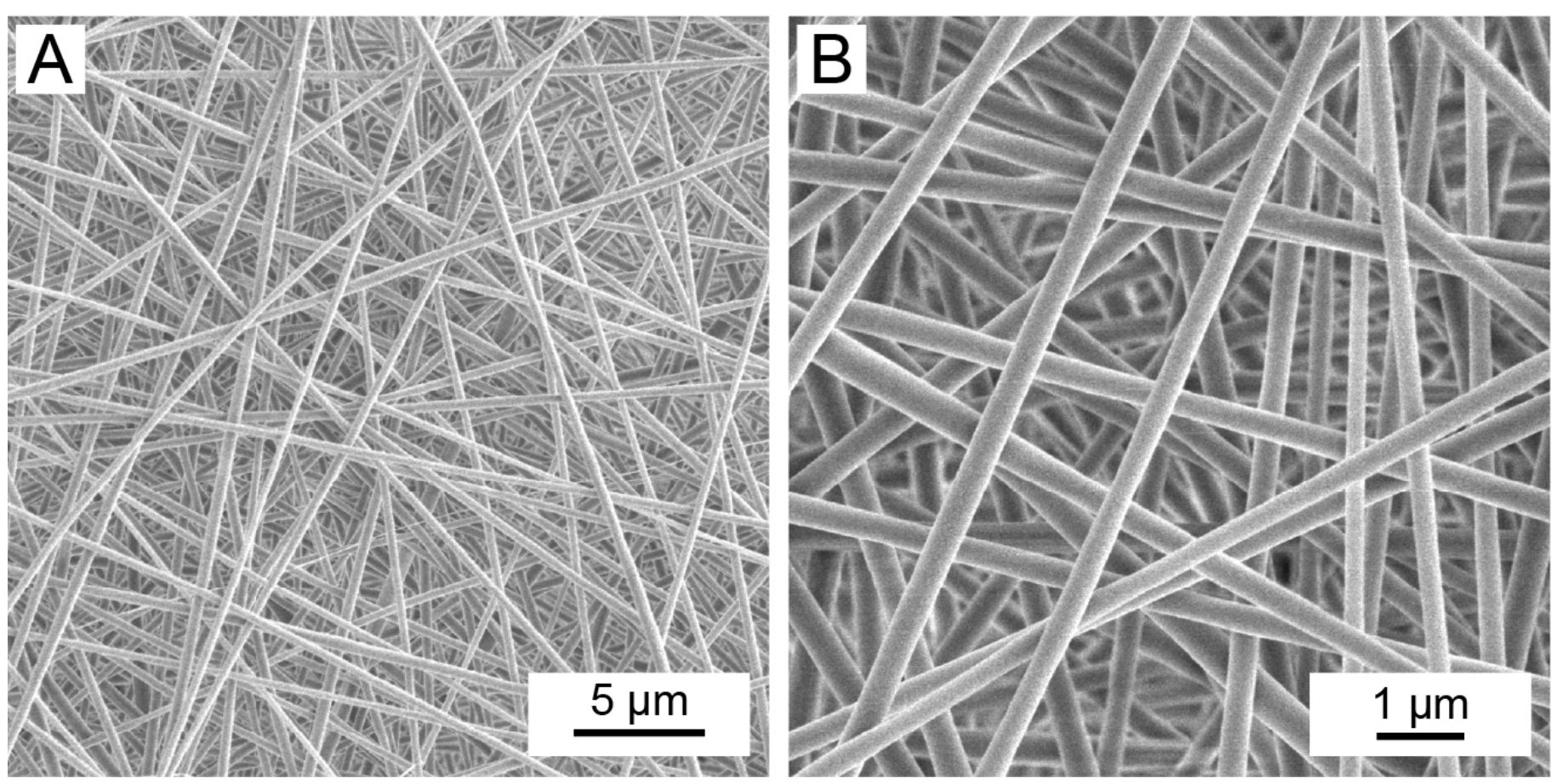

Figure S5. (A) SEM image of a mat of PCL@PAN core-shell nanofibers and (B) magnified SEM image showing fiber morphology.

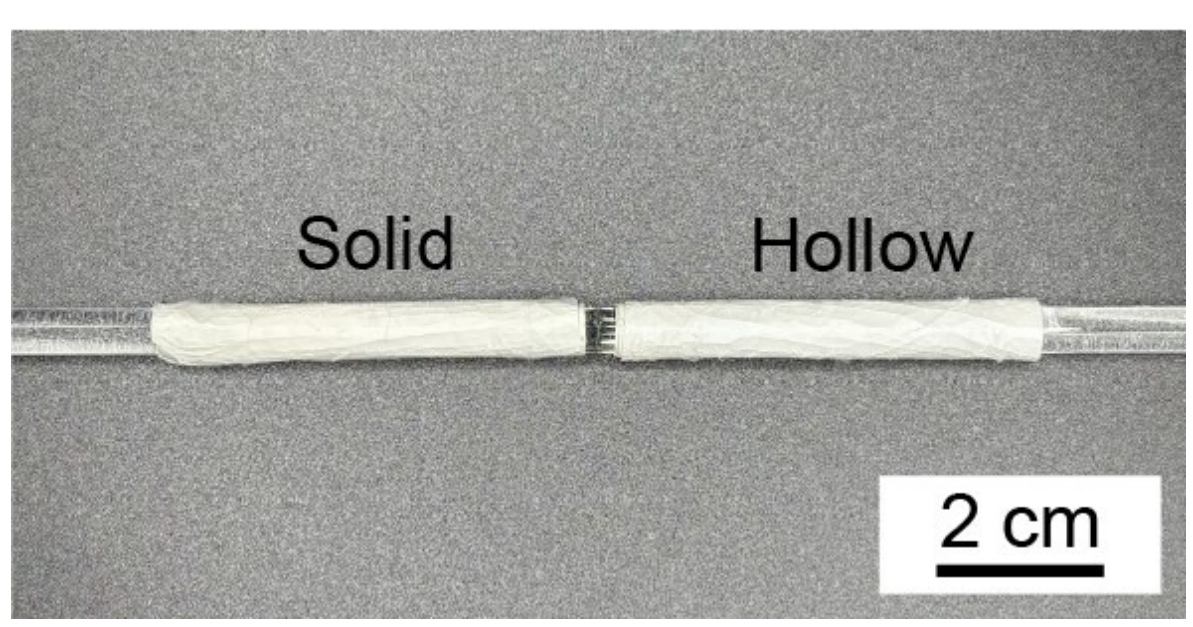

Figure S6. Photograph showing paired mats of solid and hollow nanofibers rolled around glass rods.

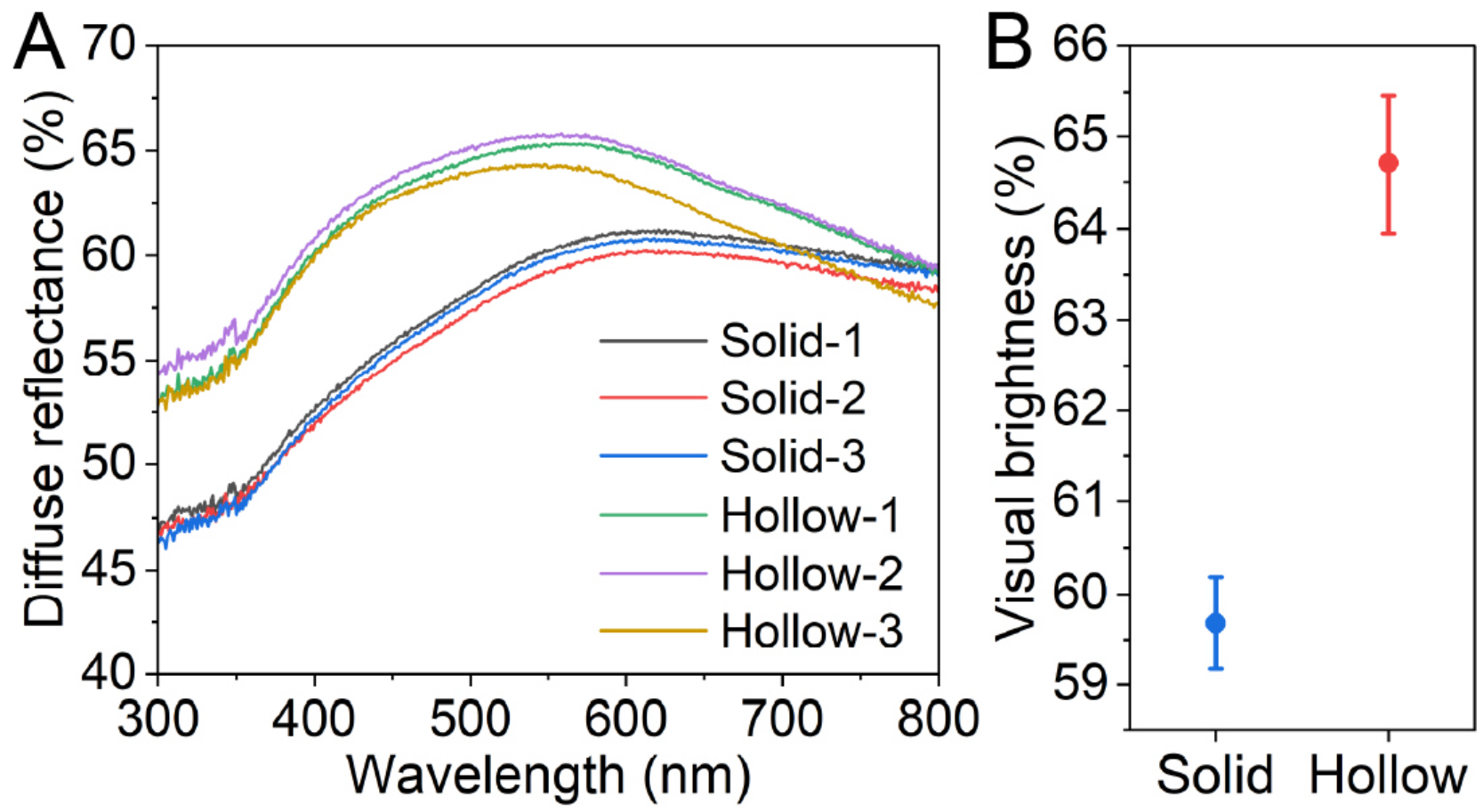

Figure S7. (A) Diffuse reflectance spectra of three solid pieces and three hollow pieces prepared from the same parent mat. (B) Mean photopic-weighted diffuse reflectance with standard deviation for comparing the visual brightness of solid and hollow samples.

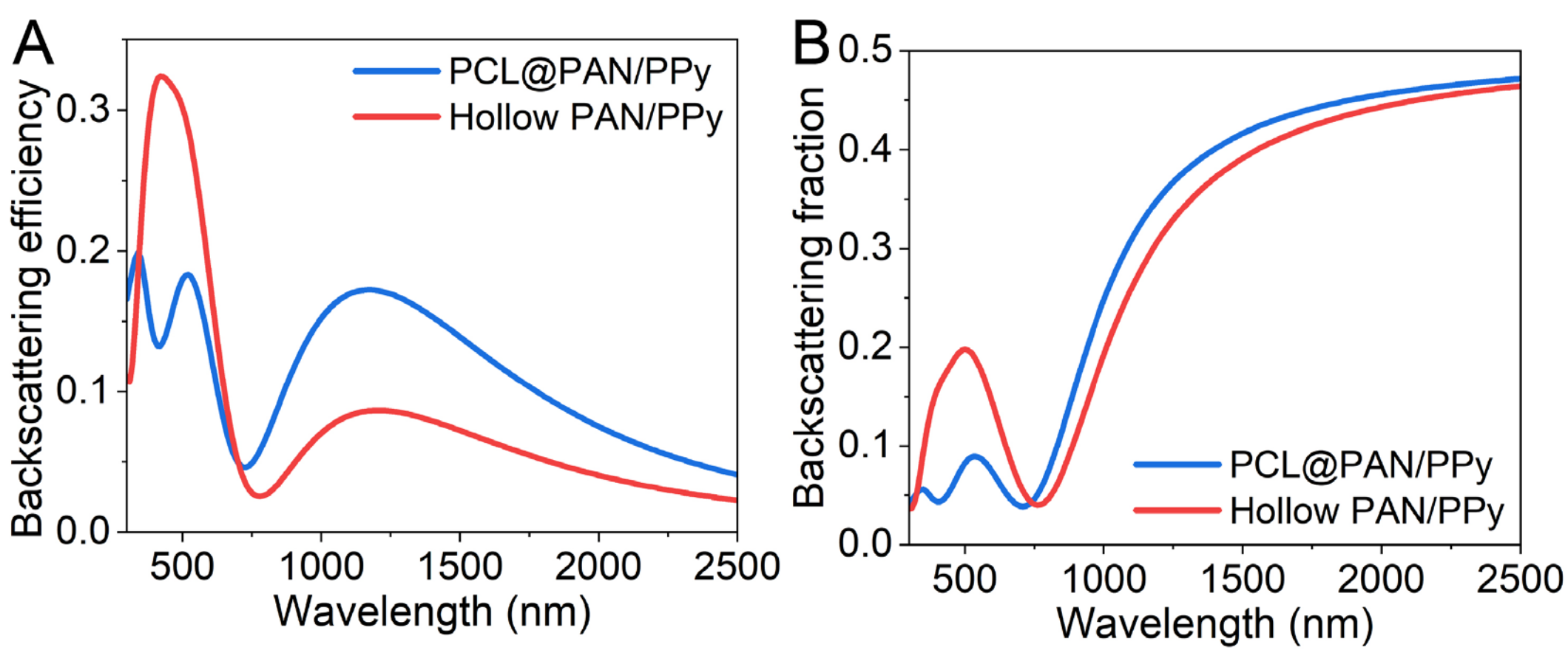

Figure S8. Simulated wavelength-dependent (A) backscattering efficiency and (B) backscattering fraction of solid PCL@PAN/PPy and hollow PAN/PPy nanofibers over 300–2500 nm.

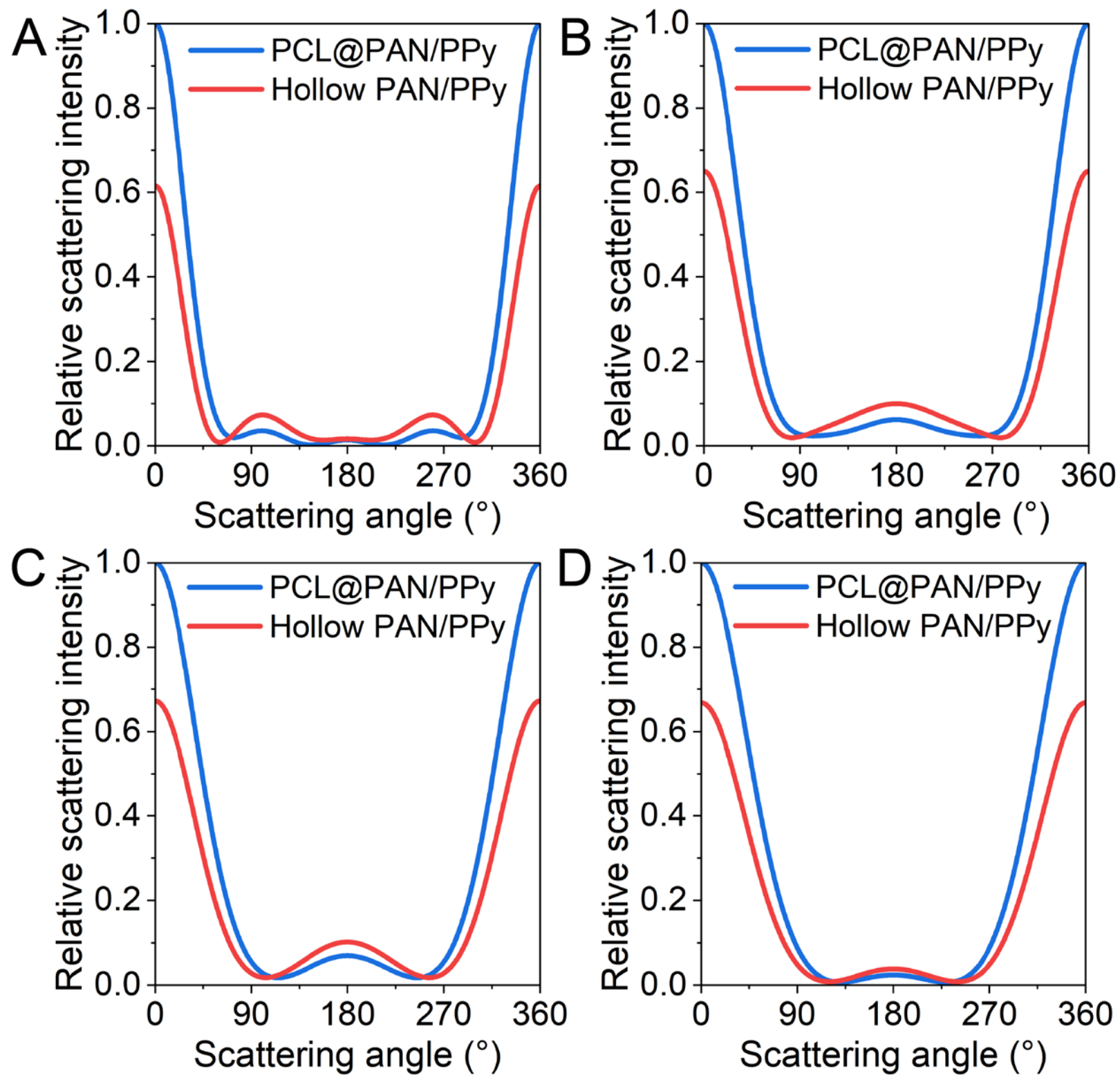

Figure S9. Simulated angular scattering distributions of PCL@PAN/PPy and PAN/PPy hollow nanofibers at wavelengths of (A) 400 nm, (B) 500 nm, (C) 600 nm, and (D) 700 nm. The scattering intensity was normalized to the maximum intensity between the solid and hollow curves at each wavelength.

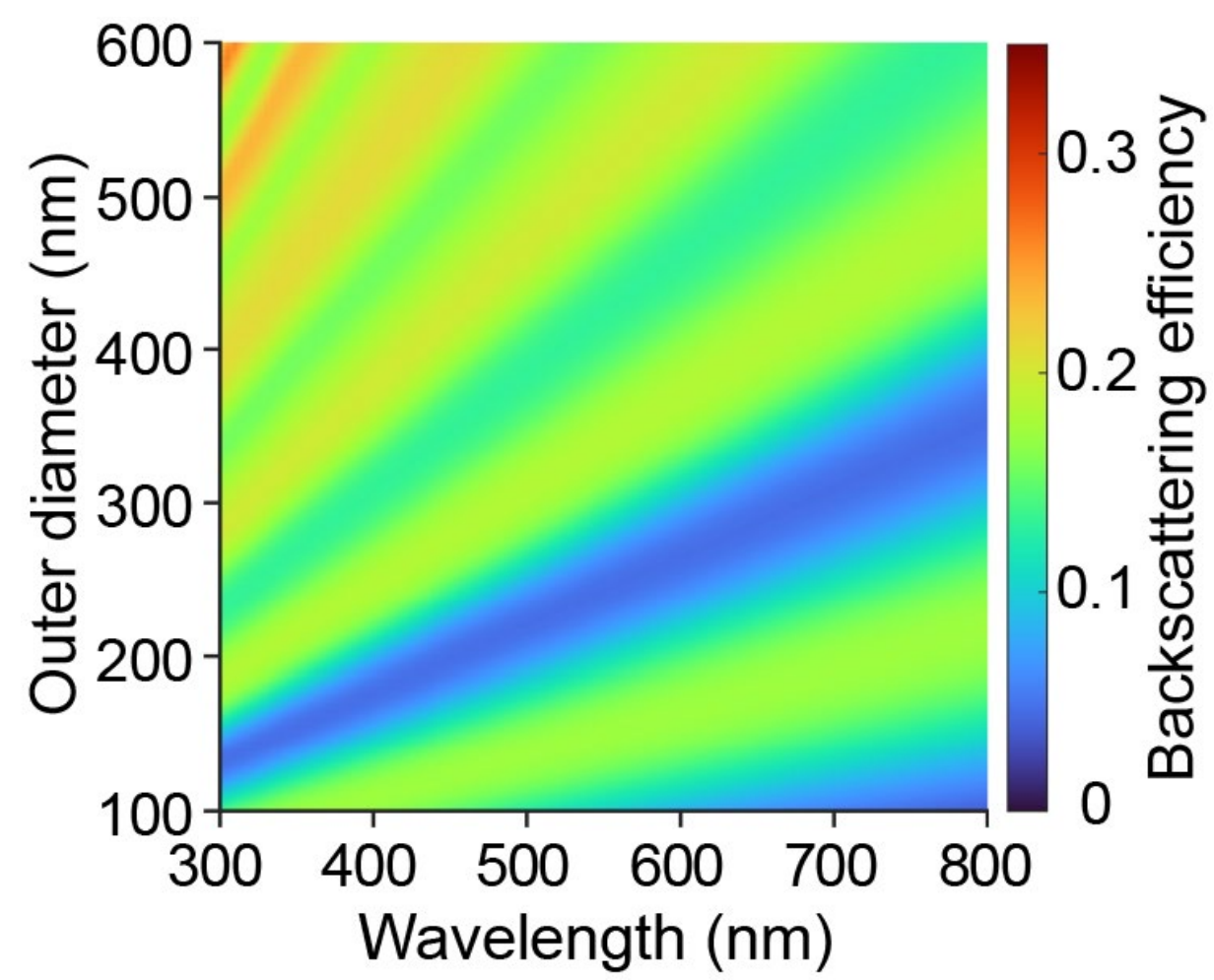

Figure S10. Two-dimensional map of the backscattering efficiency of a PCL@PAN/PPy nanofiber as a function of wavelength and outer fiber diameter, with the inner-to-outer diameter ratio fixed at 0.5.

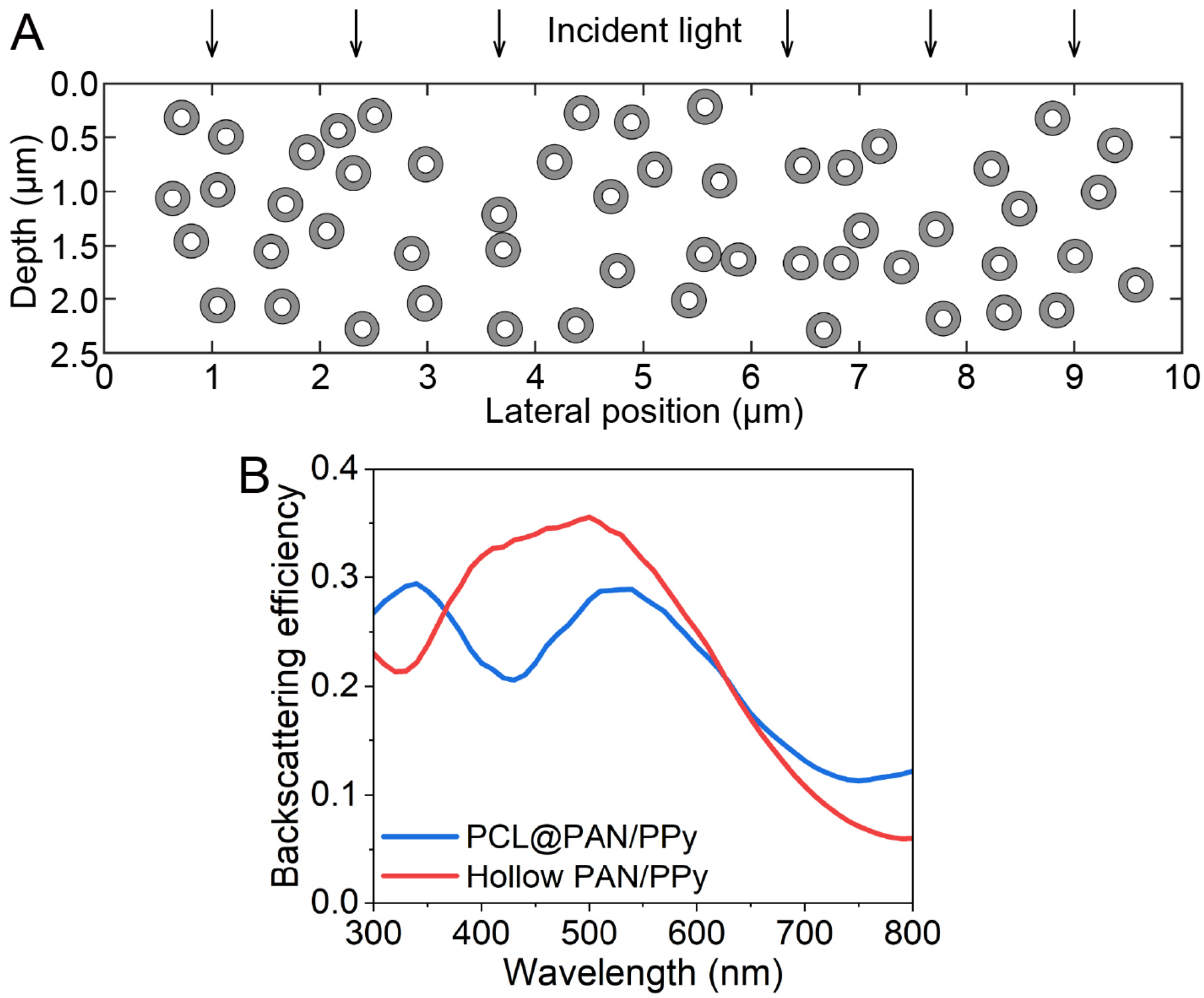

Figure S11. (A) Schematic cross-section of the simulated mat, where each ring is a nanofiber with a PCL core or an air cavity. (B) Simulated backscattering efficiency of the mats of PCL@PAN/PPy and PAN/PPy hollow nanofibers as a function of wavelength.

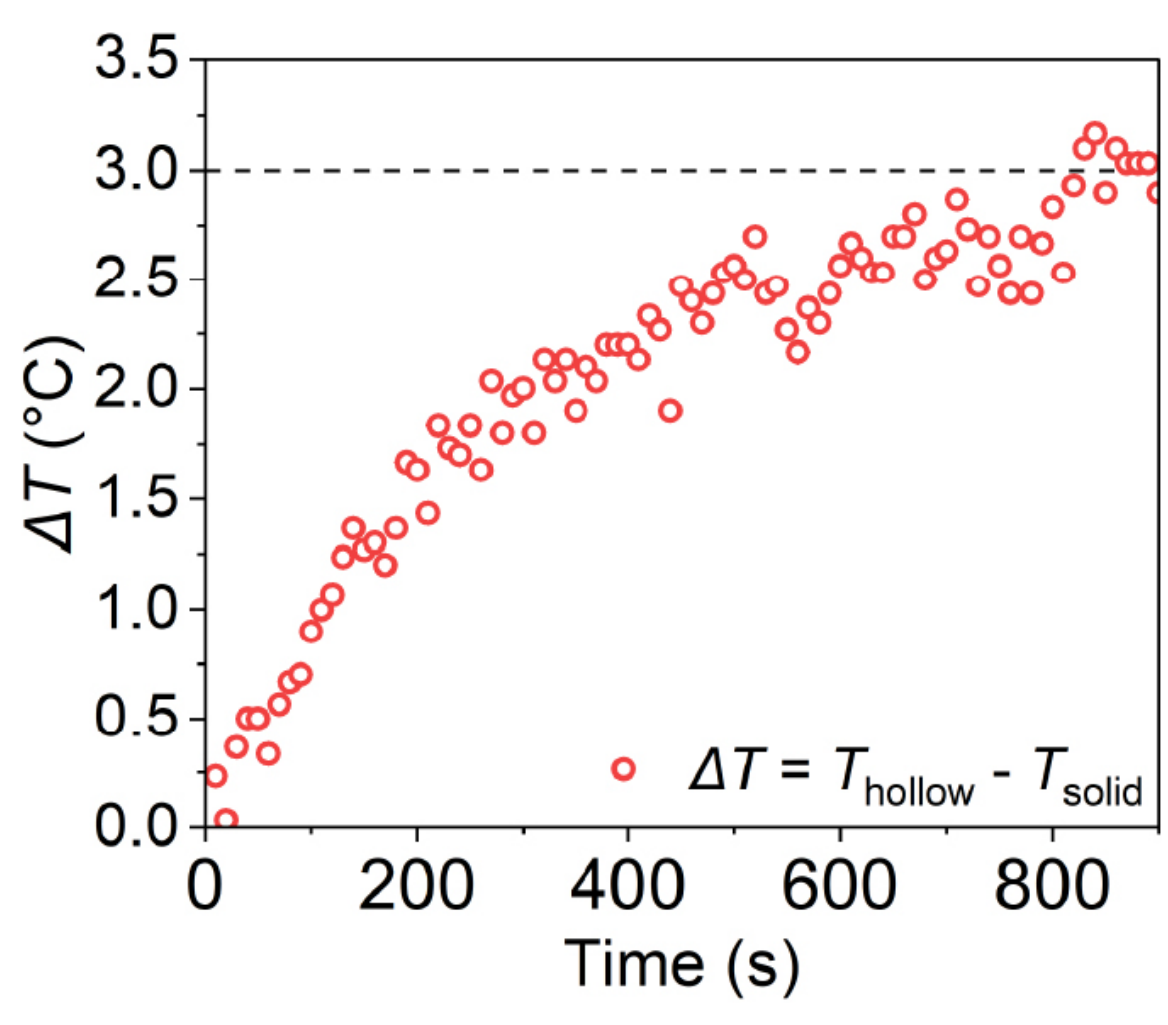

Figure S12. Time-dependent temperature difference (ΔT) between the paired mats of solid and hollow nanofibers, calculated as $\Delta T = T_{\mathrm{hollow}} - T_{\mathrm{solid}}$ using the cycle-averaged temperature profiles.

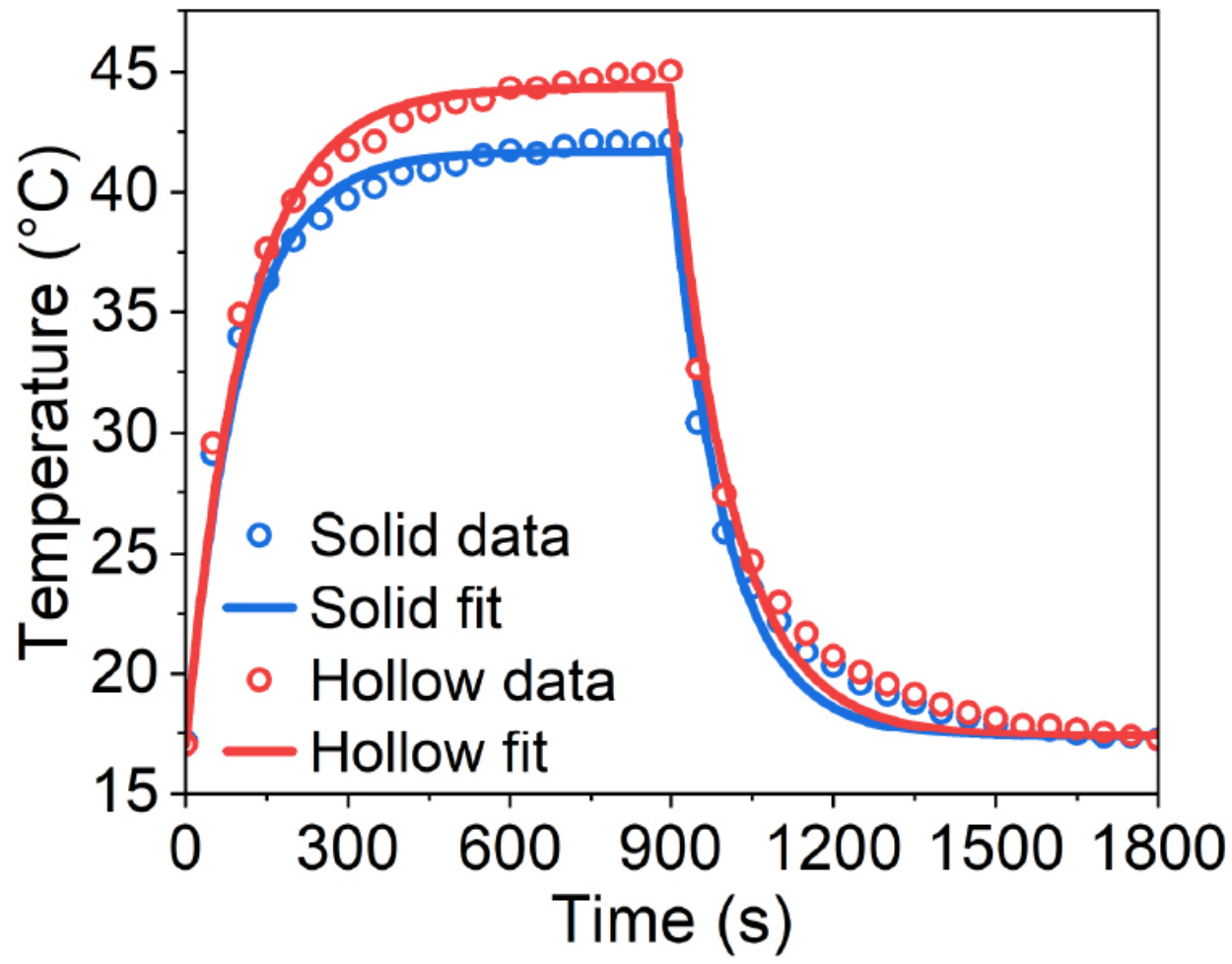

Figure S13. Cycle-averaged experimental temperature data and corresponding fits using the single-exponential thermal model for the heating and cooling processes.

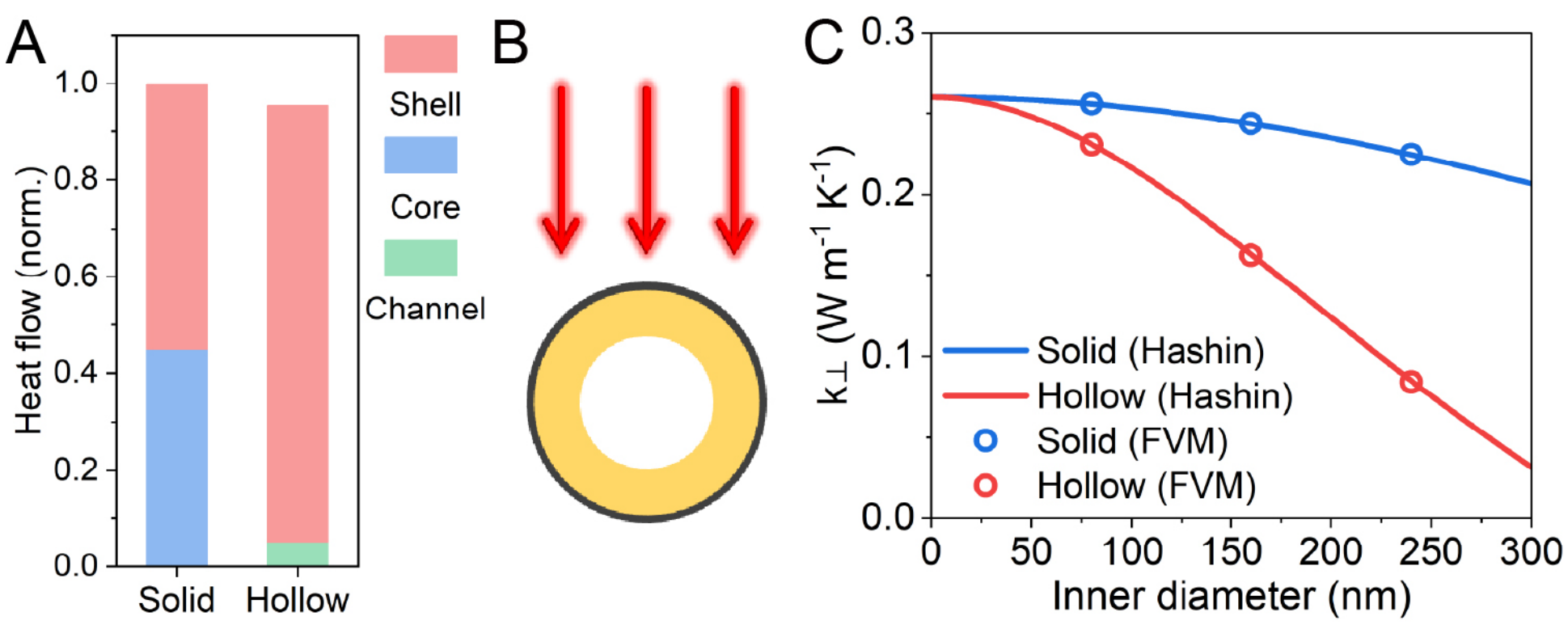

Figure S14. (A) Heat flow across the fiber, split into the contributions from the PCL core (or air channel) and the shell, and normalized to the solid fiber. (B) Schematic of the heat flow direction across a single fiber. (C) Simulated thermal conductivity across a single nanofiber, as a function of the inner diameter at a fixed outer diameter of 320 nm, for solid and hollow fibers. Curves are the analytical Hashin solution and symbols are independent calculations by FVM.

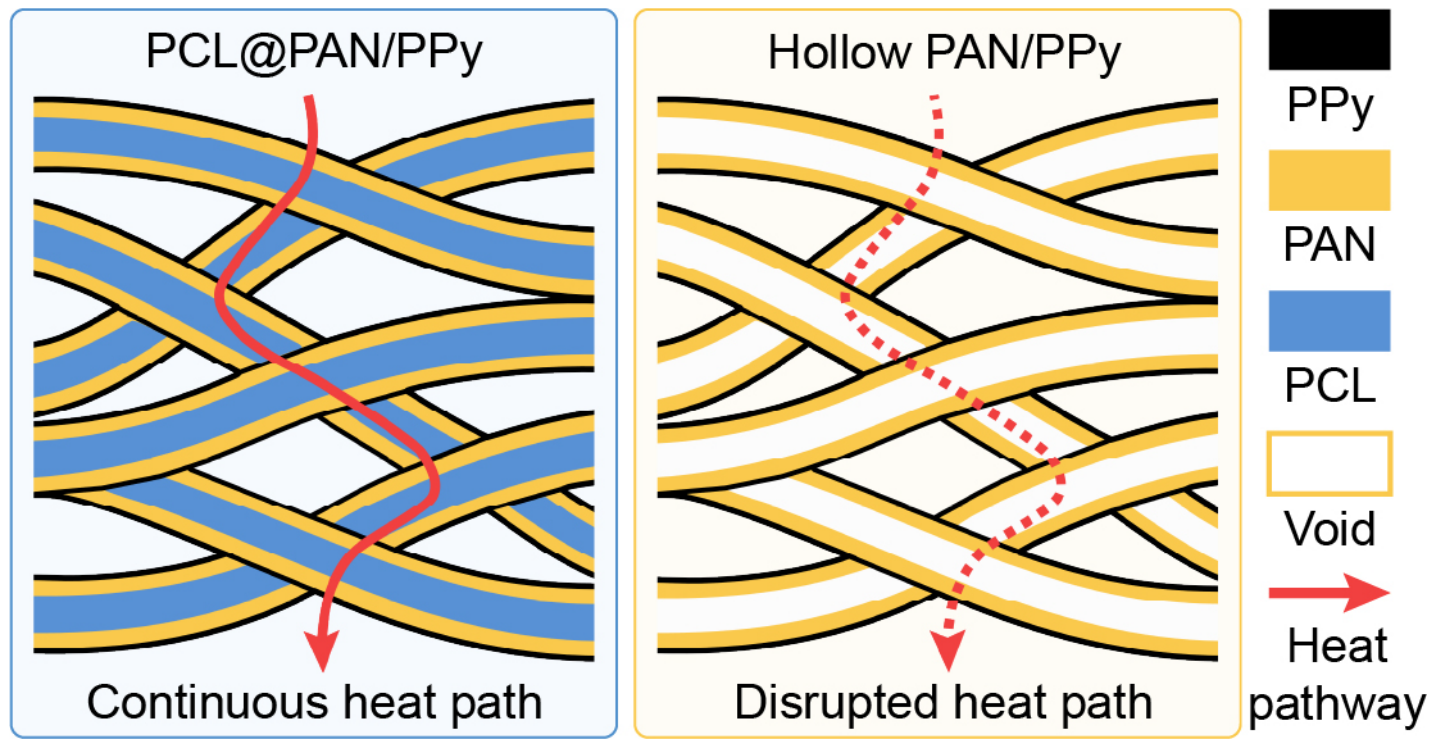

Figure S15. Schematic illustration of heat-transfer pathways in mats of solid and hollow nanofibers.

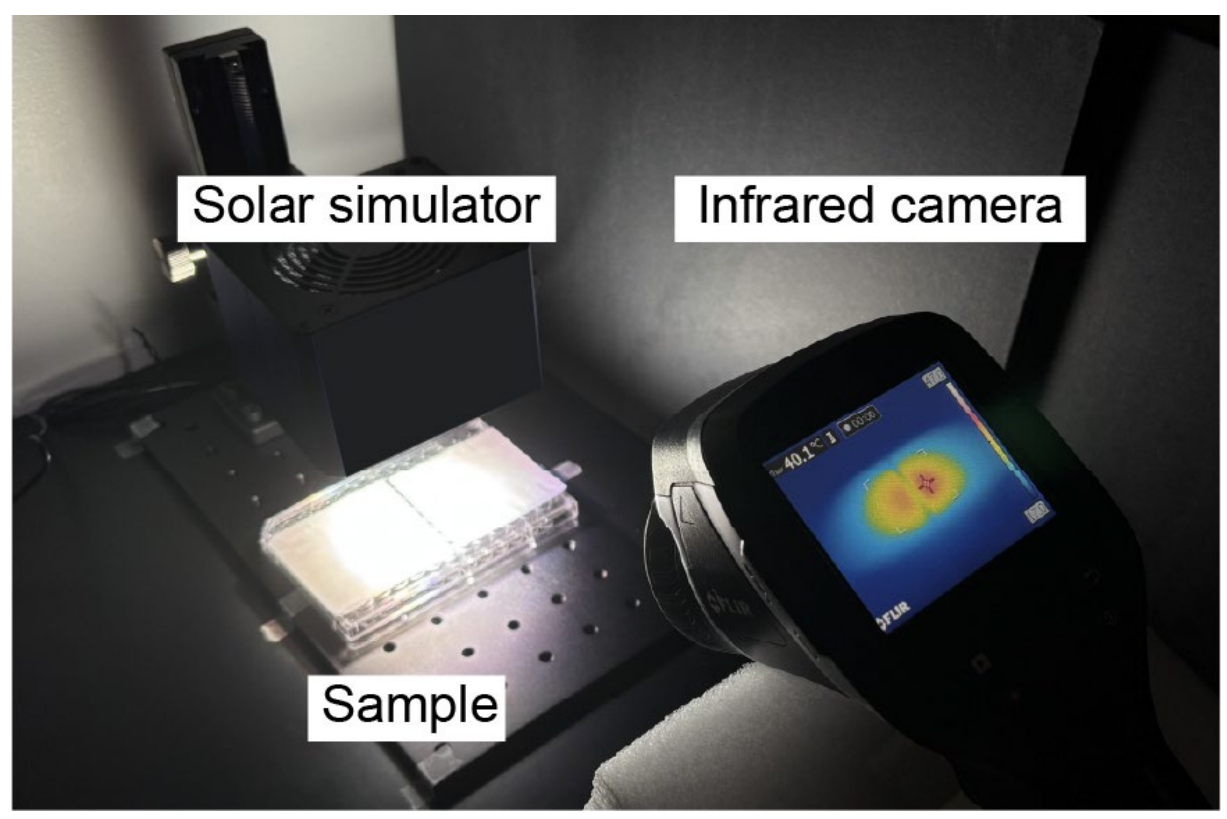

Figure S16. Photograph of the indoor side-by-side test setup, where paired mats of solid and hollow nanofibers were placed on the same substrate under 1 sun irradiation and monitored using an infrared camera.

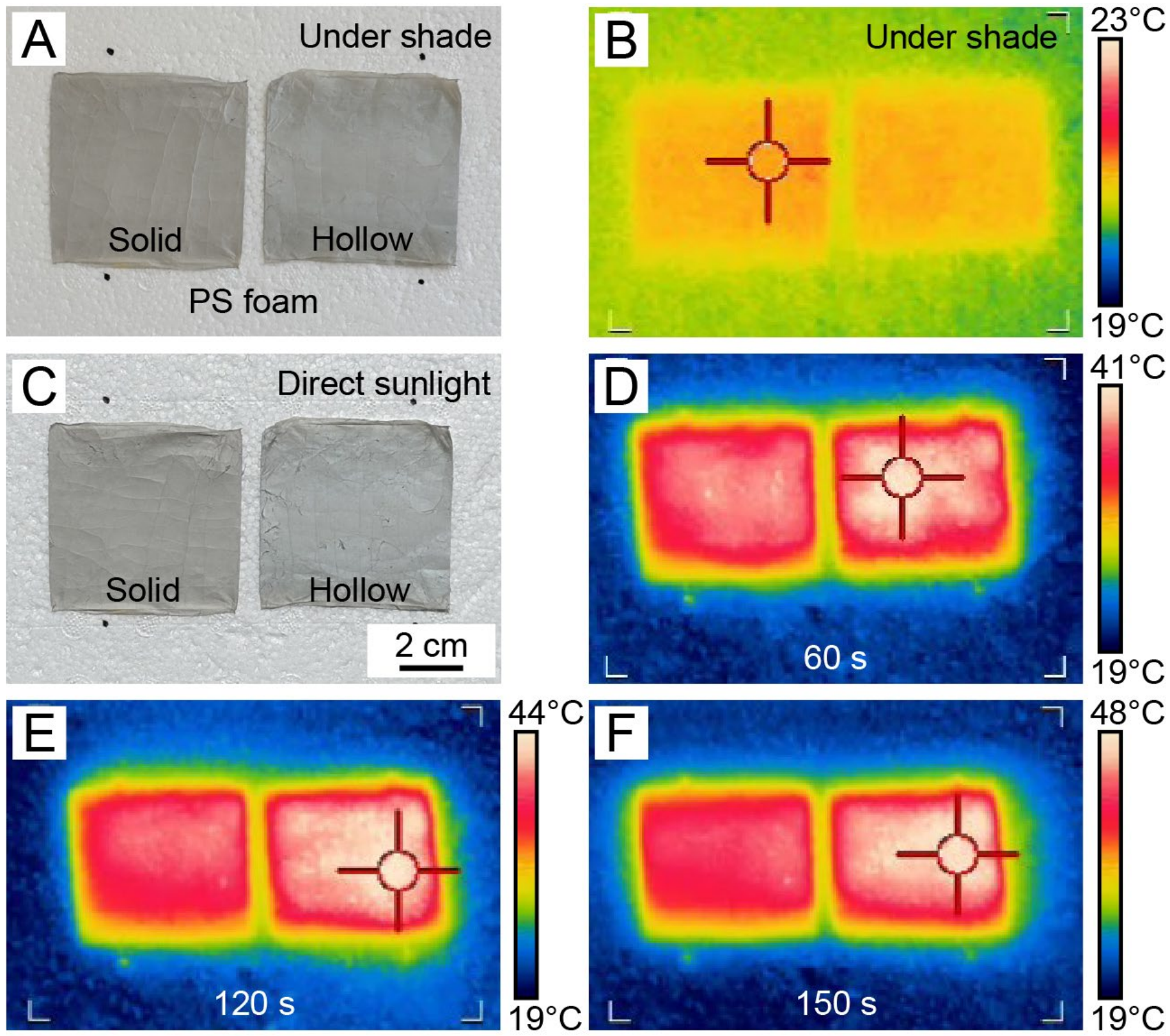

Figure S17. (A) Photograph and (B) infrared image of the paired mats of solid and hollow nanofibers on the PS foam under shade. (C) Photograph of the paired mats under direct sunlight. (D-F) Infrared thermal images of the paired mats under direct sunlight at t = 60, 120, and 150 s.

Table S1. Fitting quality of the single-exponential and substrate-coupled thermal models for mats of solid and hollow nanofibers.

Sample	Model	RMSE (°C)	R^2
Solid	Single-exponential	0.909	0.9922
Solid	Substrate-coupled	0.174	0.9997
Hollow	Single-exponential	0.967	0.9926
Hollow	Substrate-coupled	0.142	0.9998

RMSE is root-mean-square error. R^2 is coefficient of determination.

Table S2. Fitted parameters and derived quantities from the substrate-coupled thermal model for mats of solid and hollow nanofibers.

**Quantity**	**Meaning**	**Solid**	**Hollow**	**Change (%)**
$\Delta T_{s,ss}$	Steady-state surface temperature rise (°C)	25.0	27.7	+10.8
K_s/C_s	Heat-loss rate from sample surface (s^{-1})	0.0157	0.0144	−8.3
K_{sp}/C_s	Sample–substrate heat-exchange rate (s^{-1})	0.0074	0.0072	−2.7
Q_0/C_s	Initial solar-heating driving rate (°C s^{-1})	0.3928	0.3974	+1.2

Table S3. Visual brightness and temperature rise of this work and reported solar-heating textiles.

Sample	Visual brightness (%)	Temperature rise (°C)	References
PCL@PAN/PPy	59.7	25.0	This work
Hollow PAN/PPy	64.7	28.0	This work
Cs-Ny-III	69.0	24.6	[14]
RC/MXene II	63.0	20.9	[32]
RC/MXene III	40.5	27.2	[32]
RC/MXene IV	35.5	30.4	[32]
ATPLA-0.4	48.0	14.0	[33]
CWPLA-0.4	40.0	20.0	[33]
TAWT	18.0	29.0	[34]
TpPa/M	11.2	15.0	[35]
TpPa/Ag@M	8.4	25.0	[35]
PDA fabric	13.0	13.5	[36]
MXene-PDA	4.8	26.7	[36]
TFC	3.1	29.0	[37]
PFPPC-10	2.2	23.5	[38]

Visual brightness was obtained from visible-region reflectance. Reference numbers refer to the main text.
